# Altered physiological functions and ion currents in atrial fibroblasts from patients with chronic atrial fibrillation

**DOI:** 10.14814/phy2.12681

**Published:** 2016-01-26

**Authors:** Claire Poulet, Stephan Künzel, Edgar Büttner, Diana Lindner, Dirk Westermann, Ursula Ravens

**Affiliations:** ^1^ Department of Pharmacology and ToxicologyMedical Faculty Carl‐Gustav‐CarusTU DresdenDresdenGermany; ^2^ Department of General and Interventional CardiologyUniversity Heart Center Hamburg EppendorfHamburgGermany; ^3^ Imperial College LondonNational Heart and Lung InstituteLondonUK; ^4^ Biotechnology Centre DresdenTU DresdenDresdenGermany

**Keywords:** Atrial fibrillation, electrophysiology, fibroblasts

## Abstract

The contribution of human atrial fibroblasts to cardiac physiology and pathophysiology is poorly understood. Fibroblasts may contribute to arrhythmogenesis through fibrosis, or by directly altering electrical activity in cardiomyocytes. The objective of our study was to uncover phenotypic differences between cells from patients in sinus rhythm (SR) and chronic atrial fibrillation (AF), with special emphasis on electrophysiological properties. We isolated fibroblasts from human right atrial tissue for patch‐clamp experiments, proliferation, migration, and differentiation assays, and gene expression profiling. In culture, proliferation and migration of AF fibroblasts were strongly impaired but differentiation into myofibroblasts was increased. This was associated with a higher number of AF fibroblasts expressing functional Nav1.5 channels. Strikingly Na^+^ currents were considerably larger in AF cells. Blocking Na^+^ channels in culture with tetrodotoxin did not affect proliferation, migration, or differentiation in neither SR nor AF cells. While freshly isolated fibroblasts showed mostly weak rectifier currents, fibroblasts in culture developed outward rectifier K^+^ currents of similar amplitude between the SR and AF groups. Adding the K^+^ channel blockers tetraethylammonium and 4‐aminopyridin in culture reduced current amplitude and inhibited proliferation in the SR group only. Analysis of gene expression revealed significant differences between SR and AF in genes encoding for ion channels, collagen, growth factors, connexins, and cadherins. In conclusion, this study shows that under AF conditions atrial fibroblasts undergo phenotypic changes that are revealed in culture. Future experiments should be performed in situ to understand the nature of those changes and whether they affect cardiac electrical activity.

## Introduction

Cardiac fibroblasts are an essential cell population of the heart. Their role is to maintain the integrity of the tissue, thereby ensuring a proper cardiac function, for review see (Camelliti et al. 2005; Souders et al. 2009). They are primarily responsible for the homeostasis of the extracellular matrix (ECM) through the synthesis and degradation of connective tissue components. Involved in an extensive communication with their environment – through direct cell–cell or cell‐matrix connections, and via the autocrine and paracrine actions of various cytokines and growth factors – fibroblasts sense changes in mechanical and chemical signals and react appropriately to stress or injury.

Upon pathological stimuli, such as myocardial infarction, fibroblasts proliferate, and migrate to the site of injury where they differentiate into cells resembling smooth muscle, and aptly called “myofibroblasts” (Majno et al. 1971). Similarities to smooth muscle cells include an increase in the number and extent of connections to the ECM and to other cells, and the development of a fibrillar sytem of contractile proteins (Gabbiani et al. 1972). The main protein forming the myofilaments, alpha‐smooth muscle actin (*α*SMA), has been widely used as a marker of fibroblast differentiation until today.

During the healing process, myofibroblasts help to rebuild damaged tissue by producing large amounts of fibrillar collagen, replacing lost cardiomyocytes by scar tissue (Cleutjens et al. 1995). Equipped with a *de novo* contractile apparatus, they provide mechanical strength to the remodeling tissue which might reduce scarring (Gabbiani et al. 1972; Hinz et al. 2001). This reparative procedure is essential to prevent dilation and wall thinning, but can lead to pathological fibrosis if an excessive myofibroblast activity persists. Indeed, while in most tissues myofibroblasts usually undergo apoptosis, once the healing process is over (Desmoulière et al. 1995), they remain in infarct scars for years, where they continue to promote fibrosis (Willems et al. 1994).

In other organs, including lung, kidney and liver, the abnormal persistence of myofibroblasts and resulting progressive fibrosis were shown to be associated with organ failure, reviewed in (Hinz et al. 2012). Furthermore, after myocardial infarction, collagen deposition is not only found in the infarct scar, but also in noninfarcted areas of the heart (Volders 1993; Cleutjens et al. 1995), forming reactive fibrosis around cardiomyocytes that contributes to ventricular stiffness and dysfunction (Litwin et al. 1991).

Fibrosis notably plays an important role in the pathophysiology of atrial fibrillation (AF); reviewed in (Burstein and Nattel 2008). While atrial fibrosis might originate from other underlying cardiac diseases, rapid atrial pacing alone results in ECM remodeling in animal models (Li et al. 1999; Pan et al. 2007; Avitall et al. 2008), and interstitial fibrosis was found in patients with lone AF (Frustaci et al. 1997; Boldt et al. 2004). Structural remodeling indeed correlates with the development of sustained AF (Xu et al. 2004) and might participate in the maintenance of the disease, reviewed in (Yue et al. 2011). First, interstitial fibrosis impairs local conduction thereby providing a substrate for AF (Li et al. 1999). And second, fibroblasts can alter atrial electrical activity by directly coupling with cardiomyocytes through gap junctions.

Although the presence of such connections between fibroblasts and cardiomyocytes remains to be demonstrated in human tissue, functional gap junctions were observed in animal models (Camelliti et al. 2004*a* *,* *b*) and their consequences on the electrical activity of cardiomyocytes were thoroughly studied in co‐culture systems. Because of the less negative resting membrane potential (RMP) of fibroblasts, such electrotonic interactions primarily provoke the depolarization of cardiomyocytes, resulting in (1) slowing of conduction, due to the reduced number of available Na^+^ channels and which facilitates reentry (Miragoli et al. 2006; Zlochiver et al. 2008) and (2) ectopic activity when the threshold potential for activation is reached (Miragoli et al. 2007).

In this context, exploring the electrophysiological properties of fibroblasts is of primordial importance as the expression of ion channels, and the resulting changes in electrical conductance might further impair cardiomyocyte activity. Multiple ion currents have been described in variable proportions of cultured human fibroblasts, including Na^+^ currents, various K^+^ currents (delayed rectifier, inward rectifier, and Ca^2+^‐activated channels of big conductance), and chloride currents (Wang et al. 2006; Li et al. 2009; Chatelier et al. 2012). Interestingly, recent studies using a canine model of atrial fibrillation reported the remodeling of K^+^ currents under disease conditions (Wu et al. 2014; Qi et al. 2015). The objective of this study was to compare atrial fibroblasts from patients with a normal cardiac rhythm (sinus rhythm, SR) and with chronic AF to uncover any phenotypic differences, hence providing insight into the relationship between atrial fibroblasts and atrial fibrillation.

## Material and Methods

### Ethical approval

The study was approved by the ethics committee of Dresden University of Technology (No. EK790799). Each patient gave written, informed consent. Right atrial appendages were obtained from patients with SR and with chronic AF (AF >6 months) undergoing open‐heart surgery for coronary artery and/or valve disease. More details on patients are available in Table 1.

**Table 1:** Clinical parameters of the patients involved in the study

	SR	AF
Patients, *n*	58	33
Gender, m/f	50/8	28/5
Age, year	65.3 ± 1.5	70.8 ± 1.4
Body mass index, kg/m^2^	27.7 ± 0.5	29.5 ± 0.7
CAD, *n* (%)	45 (78%)	7 (21%)
MVD/AVD, *n* (%)	7 (12%)	16 (48%)
CAD + MVD/AVD, *n* (%)	6 (10%)	10 (30%)
Hypertension, *n*	54 (93%)	32 (97%)
Diabetes, *n*	23 (40%)	17 (52%)
Hyperlipidemia, *n*	50 (86%)	26 (79%)
LVEF, %	51.4 ± 1.9	48.1 ± 2.3
Digitalis, *n* (%)	3 (5%)	13 (39%)
ACE inhibitors, *n* (%)	37 (64%)	18 (54%)
AT1 blockers, *n* (%)	10 (17%)	7 (21%)
*β*‐blockers, *n* (%)	45 (78%)	28 (85%)
Ca^2+^‐antagonists, *n* (%)	13 (22%)	11 (33%)
Diuretics, *n* (%)	20 (34%)	26 (79%)
Nitrates, *n* (%)	5 (9%)	2 (6%)
Lipid‐lowering drugs, *n* (%)	47 (81%)	20 (61%)

SR, sinus rhythm; AF, chronic atrial fibrillation; CAD, coronary artery disease; MVD, mitral valve disease requiring valve replacement; AVD, aortic valve disease requiring valve replacement; LVEF, left ventricular ejection fraction; ACE, angiotensin‐converting enzyme; AT, angiotensin receptor.

### Cell culture

Cells were isolated using the outgrowth technique and enzymatic digestion. For the outgrowth method, tissue was cut in small pieces (~ 1 mm^3^) and placed in a 25 mm‐diameter petri dish with 1 mL medium consisting of DMEM (Sigma‐Aldrich, Steinheim, Germany), 10% FCS and 1% penicillin/streptomycin (Biochrom, Berlin, Germany). The medium was changed every second day, and after 3 weeks of culture, cells were trypsinized, replated at a density of 2.5 × 10^−3^ cells per cm^2^ and further cultivated for 10–15 days before analysis. In some experiments, the culture medium was supplemented with 10 *μ*mol/L tetrodotoxin (TTX) or 1 mM tetraethylammonium plus 1 mmol/L 4‐aminopyridine (TEA + 4AP). Drugs were added freshly every time the medium was changed for 2 weeks. For the enzymatic isolation, small pieces of tissue (~ 1 mm^3^) were digested with collagenase A (Sigma‐Aldrich, Steinheim, Germany), 2 mg per 100 mg of tissue in medium, for 5 h at 37°C, 5% CO_2_. Following filtration and centrifugation, the pellet was resuspended in 1 mL medium, and the cell suspension was then plated and incubated for 12 h at 37°C, 5% CO_2_ before patch‐clamp experiments.

### Migration assay

The migration capacity of fibroblasts was assessed with the Cytoselect wound healing assay (Biocat GmbH, Heildeberg, Germany) using inserts that generate a consistent gap of 0.9 mm between the cells. After 2 weeks of culture following replating, cells were trypsinized and replated in 24‐well plates containing the wound healing inserts at a density of 5 × 10^−4^ cells per well. After letting the cells attach overnight, the inserts were removed to monitor the closure of the gap. Cells were stained with DAPI or the staining solution provided with the assay 24 h after the inserts were removed. The number of migrating cells was evaluated by counting the number of nuclei within the gap.

### Gene expression analysis

Total RNA from cells was isolated using the peqGOLD Total RNA kit (PeqLab, Erlangen, Germany) according to the manufacturer's instructions. RNA was cleared off genomic DNA by DNase digestion (DNase I digest kit, PeqLab).

The GeneChip^®^ Human Gene 1.0 ST Array (Affymetrix, Santa Clara, CA, USA) comprises 36,079 transcripts. Gene ST stands for sense target – the probes on the array are selected to be distributed along the entire sequence of the transcript. First RNA integrity was checked by analyzing on the 2100 Bioanalyzer (Agilent technologies, PA, USA). For the first cycle cDNA synthesis 300 ng of total RNA was used. During the following in vitro transcription reaction cRNA was obtained and used as starting material for the second cycle cDNA synthesis. The second cycle random‐primed single‐strand DNA synthesis resulted in a product containing incorporated deoxyurinine at predefined ratios relative to thymidine (Ambion ^®^ WT Expression Kit, Ambion, Austin, Texas, USA). Subsequently, 5.5 *μ*g of this generated single‐strand DNA was fragmented with a combination of uracil DNA glucosylase (UDG) and apurinic/apyrimidinic endonuclease 1 (APE1) and then labeled using the WT terminal labeling kit (Affymetrix Inc., Santa Clara, CA, USA). The biotinylated DNA was added to a hybridization cocktail for hybridization to the Human Gene 1.0ST Array, also containing 50 pM control oligonucleotide B2, eukaryotic hybridization controls (bioB, bioC, bioD, 1.5, 5, 25 pM, respectively), according to the manufacturer's protocol (Hybridization Wash and Stain Kit, Affymetrix). The hybridization in the Affymetrix hybridization oven 640 at 45°C and 60 rpm for 16 h, the staining and washing, processed in the Fluidics Station 450 and the scanning on the GeneChip Scanner 3000 G7 system were carried out as recommended by Affymetrix (Affymetrix Inc., Santa Clara, CA, USA). The CEL files were imported to the Partek Genomics Suite 6.6 software. The data processing steps involved the background correction on the PM values, the quantile probe normalization across all arrays of the experiment as well as the log2 transformation. A mean value of 5 was set to be a detectable expression in the investigated sample. To find differentially expressed genes, analysis of variance (ANOVA) in a multifactor experiment was used (Fisher 1925). A step‐up false discovery rate (FDR) was applied to p values from the linear contrasts to determine a cutoff for significantly differentially expressed genes (Benjamini and Hochberg 1995).

### Immunostaining

For immunostaining experiments, fibroblasts were cultivated on glass coverslips. Cells were fixed with 4% formaldehyde (Thermo scientific, Waltham, USA) for 10 min, permeabilized using 0.5% Triton X‐100 for 15 min and blocked with 10% FCS (Biochrom, Berlin, Germany) in PBS for 30 min at room temperature. Incubation with primary antibodies (all from Sigma‐Aldrich, Steinheim, Germany) against vimentin (1:200), human fibroblast surface protein (hFSP, 1:200), and *α*‐smooth muscle actin (1:200) were done in blocking buffer for 2 h at room temperature. Subsequent incubation with secondary antibody anti‐mouse Cy3 (1/1000) and counterstaining of nuclei with 4′,6‐diamidin‐2‐phenylindol (DAPI; 1/1000) were performed simultaneously in blocking buffer for 1 h at room temperature.

### Electrophysiological recordings

Electrophysiological recordings were performed on freshly isolated fibroblasts and cultured fibroblasts, 10–15 days after replating. Membrane potentials and ion currents were measured 37°C with standard whole cell current‐ and voltage‐clamp techniques. ISO‐2 software (MFK, Niedernhausen, Germany) was used for data acquisition and analysis. Borosilicate glass microelectrodes had tip resistances of 2–5 MΩ when filled with pipette solution. After gigaseal formation and breaking into the cell, cell capacitance was measured using fast depolarizing ramp pulses (−55 to −50 mV, 5 ms). Series resistance and cell capacitance were electronically compensated up to 75%. All chemicals and drugs were purchased from Sigma‐Aldrich. All current–voltage relations and steady‐state inactivation curves were measured within 5 min after membrane rupture. Outward K^+^ currents and Na^+^ currents were measured using voltage‐step protocols with the following solutions (mmol/L): NaCl 150, KCl 5.4, CaCl_2_ 2, MgCl_2_ 2, glucose 11, Hepes 10 (pH 7.4, adjusted with NaOH). The pipette solution consisted of (mmol/L): NaCl 8, KCl 40, potassium aspartate 80, Tris‐GTP 0.1, Mg‐ATP 5, CaCl_2_ 2, EGTA 5, Hepes 10 (pH 7.4, adjusted with KOH). Mean parameters for steady‐state activation and inactivation curves were calculated by fitting a Boltzmann function to the data points of individual experiments: *y *= 1/(1 + exp[(*x*–V_0.5_)/k]), where V_0.5_ is the potential for half maximum activation or inactivation in mV, and k the slope factor. Nonselective ion currents were measured with a voltage‐ramp protocol and a higher concentration of 20 mmol/L KCL in the bath solution to enhance the amplitude of inward rectifier K^+^ currents. The rectifier factor (RF) was defined as the ratio of outward current to inward current measured 300 ms after and 300 ms before the reversal potential, respectively. The following drugs were diluted in bath solution and applied using a rapid solution exchanger (ALA Scientific Instruments, NY, USA): TTX (100 nmol/L or 10 *μ*mol/L), Barium (1 mmol/L), TEA (1 mmol/L), 4‐AP (1 mmol/L), Paxilline (1 *μ*mol/L), and DIDS (200 *μ*mol/L). The system supplied only bath solution during drug‐free periods and for time‐matched controls (TMC).

### Drugs and chemicals

Tetrodotoxin was purchased from Carl Roth (Karlsruhe, Germany). All other compounds were from Sigma‐Aldrich (Steinheim, Germany).

### Statistical analysis

Data are expressed as means ± SEM. In most cases, the number of experiments n represents the number of patients. For electrophysiological measurement, *n* = number of cells/number of patients. Statistical comparison between means was made using two‐tailed paired or unpaired Student's *t*‐test. *P* < 0.05 was considered statistically significant. Analysis of variance (ANOVA) was applied for analysis of gene expression.

## Results

### Characterization of SR and AF cultured fibroblasts

**Figure 1:**
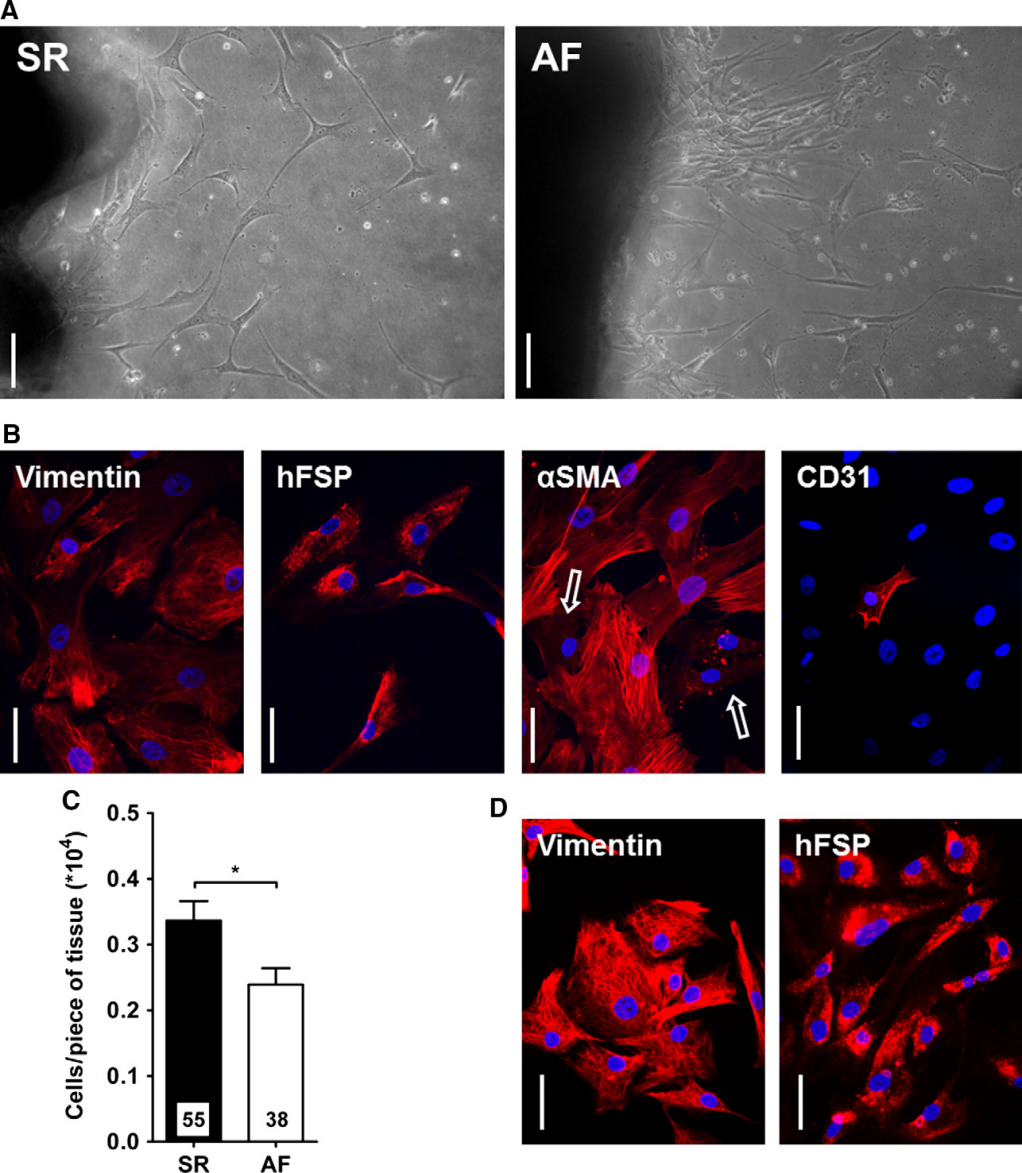
Characterization of cultured fibroblasts. (A) Fibroblasts growing out of explants from SR and AF samples after 1 week of culture. Scale bar: 100 *μ*m. (B) Staining for the expression of vimentin, human fibroblast surface protein (hFSP), alpha‐smooth muscle actin (*α* SMA) and CD31 in AF fibroblasts before replating. Nuclei are stained in blue with DAPI. Cells expressing *α* SMA‐positive myofilaments are easily distinguished from negative cells (arrows). Scale bar: 50 *μ*m. (C) Cell count after 3 weeks of culture. The number of cells was normalized to the number of tissue pieces. *P < 0.05, unpaired Student's *t*‐test. (D) Staining for Vimentin and hFSP in AF fibroblasts after replating. Nuclei are stained in blue with DAPI. Scale bar: 50 *μ*m.

Fibroblasts growing out of explants could be observed after 3–4 days of culture in both groups. The cells had the stellar shape typical of fibroblasts and no morphological difference between SR and AF cells was noted (Fig. 1A). After 2– 3 weeks of culture, most cells expressed vimentin (SR: 161/177, 92.4 ± 3.8%, *n* = 3; AF: 234/249, 89.4 ± 7.7%, *n* = 4) and human fibroblast surface protein (hFSP; SR: 136/143, 95.5 ± 2.4%, *n* = 3; AF: 174/185, 94.2 ± 1.8%, *n* = 4), confirming their fibroblast phenotype (Fig. 1B). A fraction of cells expressed *α*SMA‐positive myofilaments, (SR: 810/1414, 56.0 ± 4.4%, *n* = 6; AF: 744/1211, 59.9 ± 5.0%, *n* = 7) suggesting their differentiation into myofibroblasts. We found a few isolated cells positive for the endothelial marker CD31 (SR: 24/585, 3.8 ± 0.8%, n = 3; AF: 18/714, 1.8 ± 1.6%, n = 4) which implies that the rare endothelial cells that migrated out of the tissue did not proliferate, and further confirms that the cell population consisted mostly of fibroblasts. A striking difference between SR and AF samples was the number of cells obtained after 3 weeks of culture (Fig. 1C). AF biopsies indeed yielded significantly less cells than SR samples (Number of cells/number of tissue pieces, × 10^4^, SR: 0.33 ± 0.04, *n* = 34; AF: 0.20 ± 0.02, *n* = 18; *P* < 0.05).

**Figure 2:**
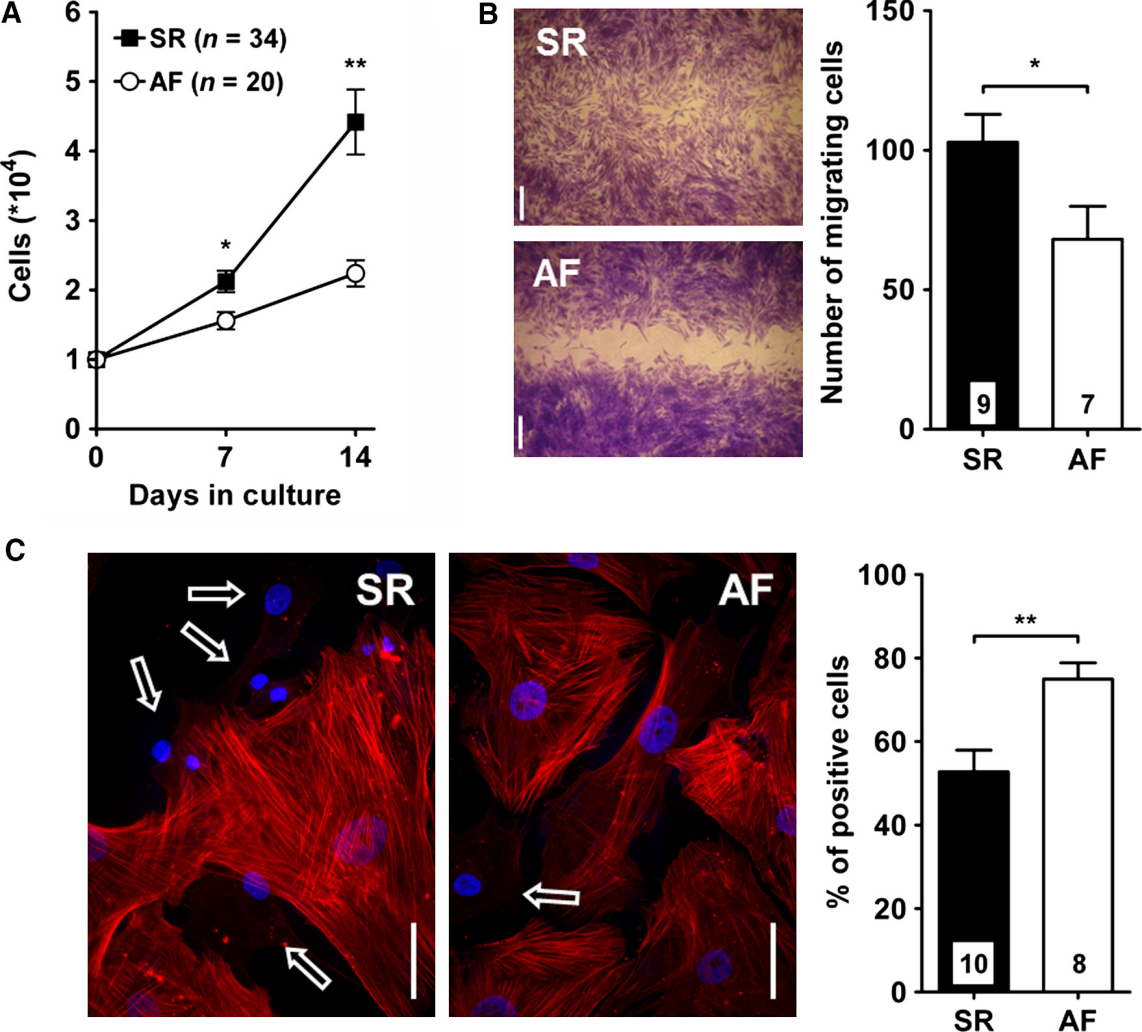
Proliferation, migration, and differentiation of cultured fibroblasts. (A) Cells were counted after 7 and 14 days of culture following replating (SR,* n *= 34; AF,* n *= 20). (B) Migration was assessed using a wound healing assay (see Material and Methods). Left panel: example of the gap closure after 24 h. The graph summarizes the number of cells closing the gap. (C) Differentiation of fibroblasts into myofibroblasts was evaluated by staining the cells for *α*‐smooth muscle actin (*α* SMA). Left panel: cells expressing *α* SMA‐positive filaments are easily distinguished from negative cells (arrows). Right panel: The graph summarized the fraction of cells positive for *α* SMA. *P < 0.05, **P < 0.01, unpaired Student's *t*‐test.

After fibroblasts were replated, 99–100% of SR and AF cells expressed vimentin (SR: 292/292: AF: 253/255, *n* = 4 in both groups) and hFSP (SR: 282/282; AF: 234/234, *n* = 4 in both groups, Fig. 1D). At this stage of culture, we observed important differences between the two groups (Fig. 2). First the proliferation of AF fibroblasts was much lower than that of SR cells (number of cells after 14 days, × 10^4^, SR: 4.4 ± 0.5, *n* = 34; AF: 2.2 ± 0.2, *n* = 20; *P* < 0.01). Second, AF fibroblasts also showed an impaired capacity for migration (number of migrating cells after 24 h, SR: 102.9 ± 10.0, *n* = 9; AF: 68.1 ± 11.8, *n* = 7; *P* < 0.05). And finally, a larger number of AF fibroblasts differentiated into myofibroblasts (SR: 542/1065, 52.8 ± 5.2; AF: 500/680, 75.0 ± 3.9; *P* < 0.01).

**Figure 3:**
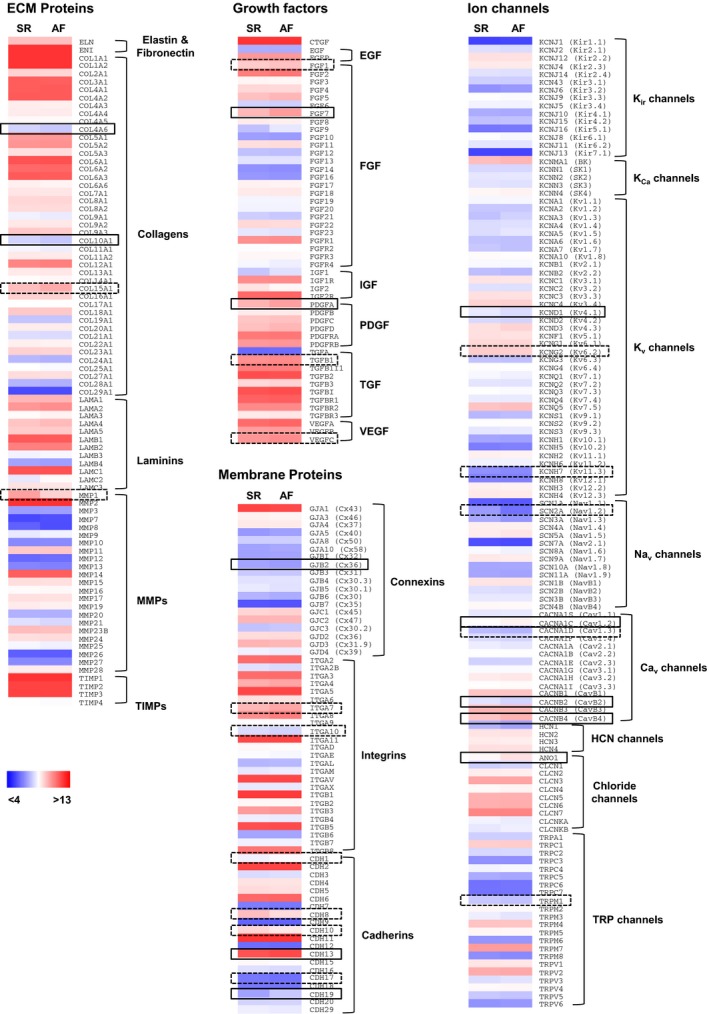
Gene expression of major ECM components, growth factors, membrane proteins, and ion channels in cultured fibroblasts. Gene expression was quantified in SR (*n *= 8) and AF (*n *= 5) fibroblasts 2 weeks after replating using an Affymetrix GeneChip array (see Material and Methods). Colors represent log2‐transformed expression levels from low (blue) to high (red) levels. Boxes denote significant (solid line, *P* < 0.05) and nonsignificant (dotted line, 0.05 <  *P *<* *0.1) differences between SR and AF. Absolute expression levels, fold change, and *P* values are given in Table S1.

SR and AF fibroblasts showed overall similar expression levels of extracellular matrix constituents and growth factors (Fig. 3 and Table  S1). They expressed high levels of fibronectin and various members of the collagen family, mostly of type I, III, IV, V, and VI. Differences between the two groups are noteworthy, such as a significant lower expression of COL4A6 and COL10A1 in AF fibroblasts but a tendency for higher COL15A1 levels. We found high levels of matrix metalloproteinase (MMP) 1, 2, and 14 and tissue Inhibitor of metalloproteinase (TIMP) 1, 2, and 3 with a tendency for reduced MMP1 expression in AF fibroblasts. We found, in both SR and AF cells high levels of connective tissue (CTGF), fibroblast growth factors (FGF) 1, 2, 5, and 7, as well as FGF‐receptor 1. Most members of the platelet‐derived growth factor (PDGF), transforming growth factor (TGF)‐Beta, and vascular endothelial growth factor (VEGF) families were also highly expressed in both groups. AF fibroblasts showed a significant upregulation of FGF7 and PDGFA and a tendency for higher levels of FGF1, TGF*β*1, and VEGFC. Finally while most integrins (ITG) and cadherins (CDH) were similarly expressed in AF and SR cells, CDH13 and CDH19 were significantly upregulated in the AF group. We also observed a tendency for higher levels of ITGA7 and CDH1 and lower levels of ITGA10, CDH8, CDH10, and CDH17. The cardiac‐specific gap junction protein connexin 43 (Cx43) was highly expressed in both groups. Cx26 was significantly downregulated in AF fibroblasts.

### Ion currents in fresh and cultured fibroblasts

**Figure 4:**
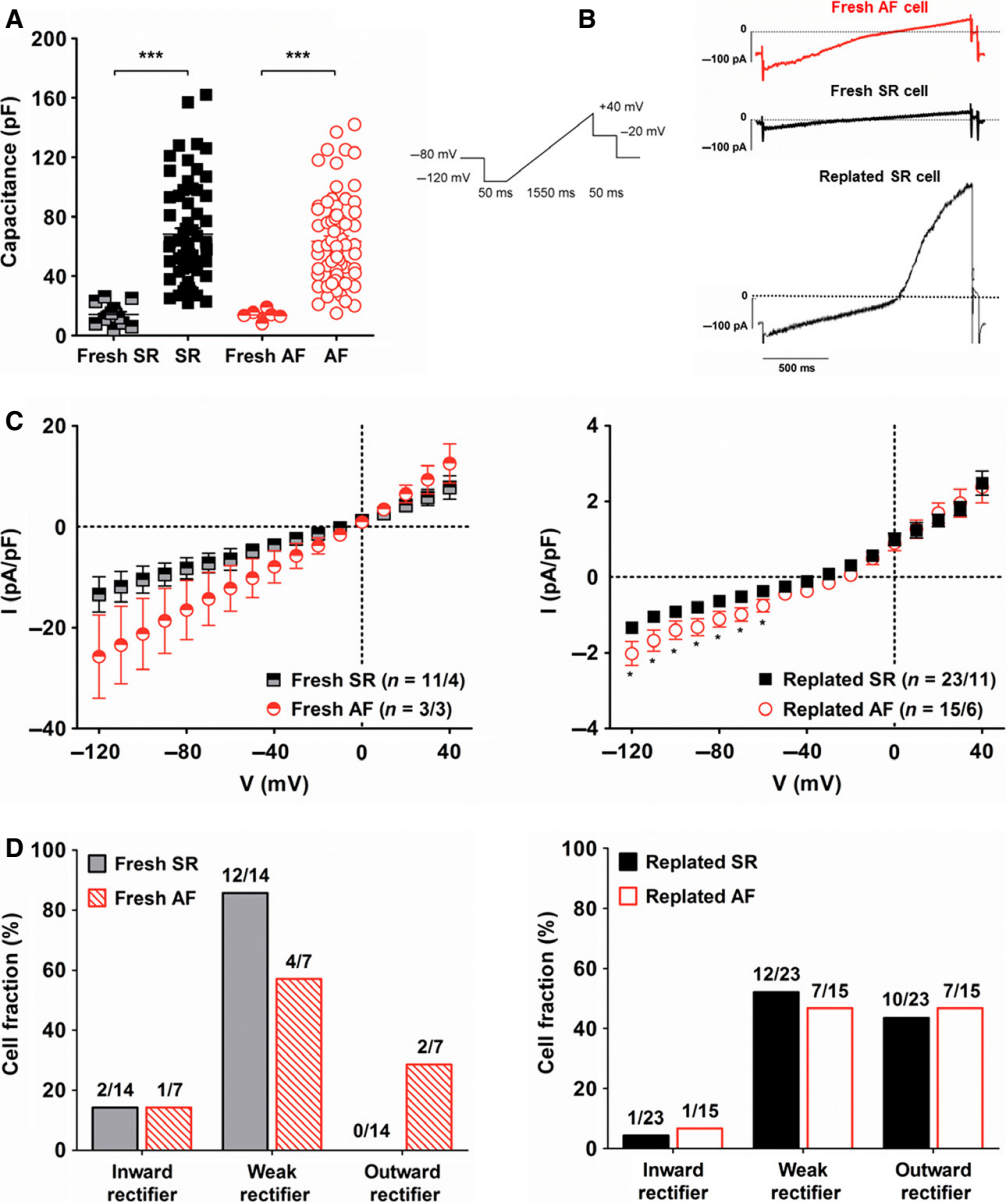
Ion currents in freshly isolated and cultured fibroblasts. (A) Capacitance was measured in freshly isolated fibroblasts from four SR patients and three AF patients; and in replated fibroblasts from 11 SR patients and 10 AF patients. (B) Left panel: voltage‐ramp protocol for current activation. Right panel: Example of different types of currents recorded in AF (red) and SR (black) fresh or replated fibroblasts. [K^+^]_o_ = 20 mmol/L. (C) Current–voltage relationship of currents measured in freshly isolated (left) and replated fibroblasts (right). (D) Fraction of freshly isolated (left) and replated fibroblasts (right) cells exhibiting inward rectifier, weak rectifier and outward rectifier current [defined by rectifier factors (see Material and Methods) of <0.8, 0.8 to <1.6, and >1.6, respectively].

In order to understand the impact of culture conditions on the phenotype of our cells, we measured ion currents in freshly isolated fibroblasts, 12 h after isolation, and cultured fibroblasts, 10–15 days after the first passage (Fig. 4).

As shown by cell capacitance measurements, fresh fibroblasts were much smaller than replated cells (Fig. 4A) with a capacitance of 14.2 ± 1.8 pF (*n* = 14) for fresh SR cells vs. 68.1 ± 4.0 pF (*n* = 66) for replated SR cells (*P* < 0.0001); and 14.6 ± 1.4 pF (*n* = 7) for fresh AF fibroblasts versus 63.5 ± 3.3 pF (*n* = 77) for replated AF cells (*P* < 0.0001).

The amplitude of inward currents in fresh fibroblasts was more variable and significantly larger than in replated cells (Fig. 4C). At −120 mV, current amplitude was −13.4 ± 3.5 pA/pF (*n* = 14/4) in fresh SR cells versus −1.3 ± 0.1 pA/pF (*n* = 23/11) in replated SR fibroblasts (*P* < 0.0001); and −25.7 ± 8.2 pA/pF (*n* = 7/4) in fresh AF cells versus −2.0 ± 0.3 pA/pF (*n* = 15/6) in replated AF fibroblasts (*P* < 0.001). Inward current amplitude in fresh AF cells was larger than in fresh SR cells but this difference was not significant. In replated fibroblasts, however, inward currents in AF cells were significantly larger than in SR cells at voltages from −120 to −60 mV.

Barium, a nonselective blocker of inward rectifier K^+^ (Kir) channels did not affect inward current amplitude in replated fibroblasts (Table 2). We could detect transcripts for the I_K1_ subunits, Kir2.2 and Kir2.3 in both replated SR and AF cells (Fig. 3 and Table S1), however, as these channels are sensitive to Ba^2+^, they are unlikely to be involved in the currents observed.

**Table 2:** Effect of various drugs on ion currents

	SR	AF
Inward K+ currents		
TMC	99.9 ± 3.7 (7/4)	91.9 ± 4.8 (6/2)
Barium, 1 mmol/L	104.7 ± 1.7 (6/3)	92.2 ± 10.4 (4/3)
Outward currents		
TMC	100.1 ± 4.7 (7/3)	97.2 ± 3.1 (6/3)
TEA, 1 mmol/L	83.2 ± 2.5 (7/4)**	86.6 ± 4.7 (11/3)
4‐AP, 1 mmol/L	86.3 ± 2.9 (5/3)*	94.8 ± 2.1 (11/3)
Paxilline, 1 *μ*mol/L	97.8 ± 6.3 (6/3)	97.2 ± 5.2 (4/2)
DIDS, 200 *μ*mol/L	91.5 ± 5.2 (4/3)	96.5 ± 8.6 (5/2)

**Figure 5:**
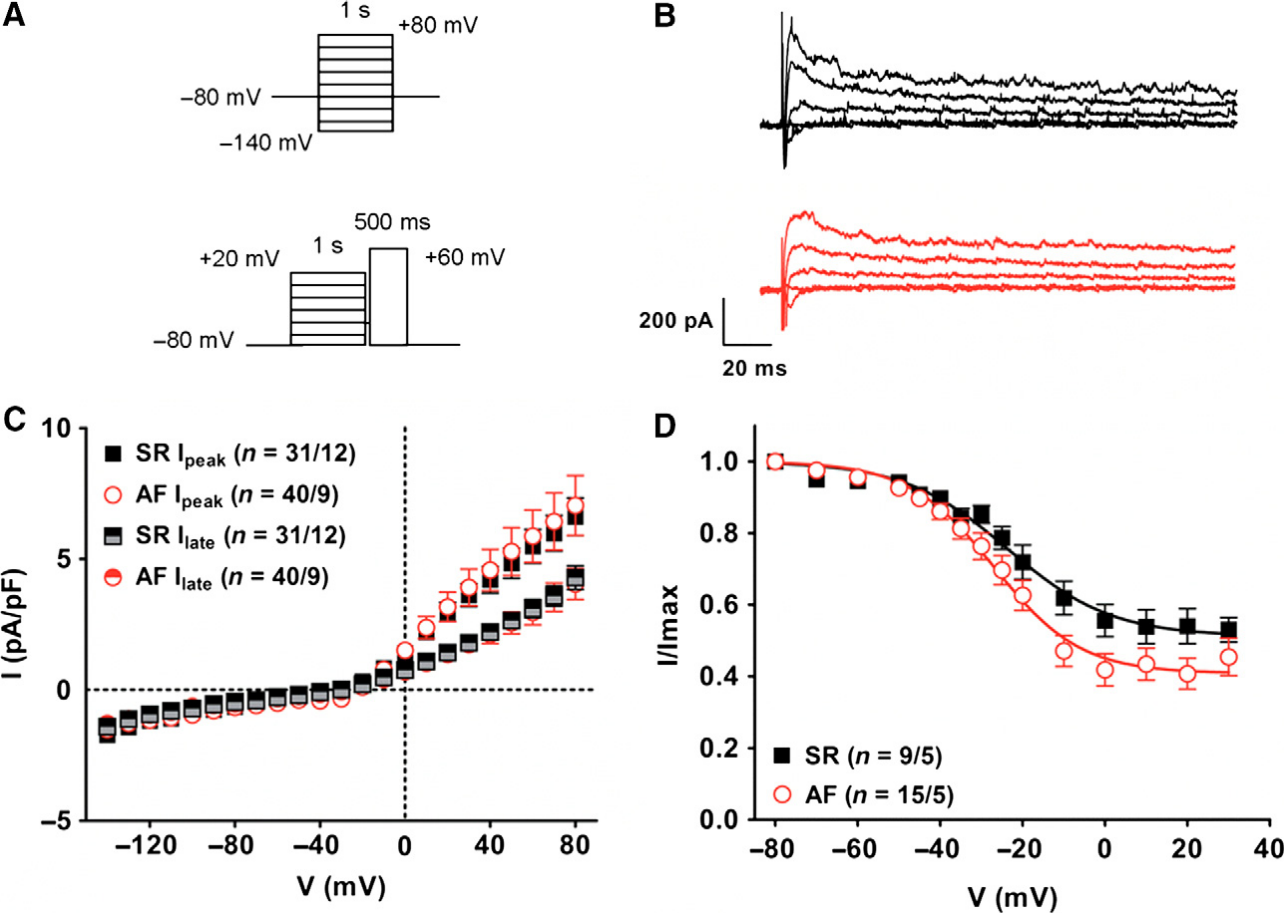
Outward rectifier currents in cultured fibroblasts. (A) Voltage‐step protocols for current activation (top) and steady‐state inactivation (bottom). (B) Examples of currents recorded at different potentials in a SR (black) and AF (red) fibroblast. [K^+^]_o_ = 5.4 mmol/L. (C) Current–voltage relationship of outward peak and late currents. (D) Steady‐state inactivation of outward peak currents. A Boltzmann function was fitted to the data points (see Material and Methods). Fitting parameters are given in Table 3.

Inward currents were measured at −120 mV using a voltage‐ramp protocol and high external K+ (see Fig. 4). Outward currents were measured at +60 mV using a voltage‐step protocol and low external K+ (See Fig. 5). Data are given as percentage of predrug control.

*P < 0.05; **P < 0.001, compared to TMC, unpaired Student's *t* test.

Under these experimental conditions, small outward currents could also be observed, activating at 0 mV in fresh cells and ‐20 mV in replated cells. At +40 mV, current amplitude was 7.8 ± 2.3 pA/pF (*n* = 14/4) in fresh SR cells versus 2.5 ± 0.3 pA/pF (*n* = 23/11) in replated SR fibroblasts (*P* < 0.01); and 12.5 ± 3.9 pA/pF (*n* = 7/4) in fresh AF cells versus 2.4 ± 0.4 pA/pF (*n* = 15/6) in replated AF fibroblasts (*P* < 0.01).

To further characterize ion currents measured in fresh and replated fibroblasts, we defined the rectifier factor (RF) as the ratio of outward current to inward current (see Material and Methods) and distinguished three categories: inward rectifier (RF < 0.8), weak rectifier (0.8 <  RF < 1.6), and outward rectifier (1.6 <  RF). We found that while ion currents in most fresh cells were weak rectifiers, outward rectification was observed in a large fraction of replated SR and AF fibroblasts (Fig. 4D).

### Outward potassium currents in cultured fibroblasts

Outward rectifying currents were then further characterized in replated SR and AF fibroblasts using a voltage‐step protocol and a low external K^+^ concentration (Fig. 5). We measured fast activating, slowly inactivating currents in both SR and AF fibroblasts (Fig. 5B). These currents activated around −20 mV (Fig. 5C) and mean peak current density was, at +80 mV, 6.6 ± 0.7 pA/pF (*n* = 31/12) for SR and 7.0 ± 1.1 (*n* = 40/9) for AF (n.s.). Late current density at +80 mV was 4.3 ± 0.5 pA/pF (*n* = 31/12) for SR and 4.0 ± 0.6 (*n* = 40/9) for AF (n.s.).

Steady‐state inactivation curve is shown in Fig. 5D. The parameters from curve fitting are summarized in Table 3. The steady‐state inactivation curve of AF cells was slightly shifted toward more negative potentials; however, this difference failed to reach the level of statistical significance. In both groups a comparable fraction of current did not inactivate.

**Table 3:** Potassium and sodium current parameters

	SR	AF	*P*
I_K_ parameters			
ss inactivation V_0.5_, mV	−23.0 ± 1.9	−28.6 ± 1.8	0.053
K	11.3 ± 1.9	8.4 ± 0.7	0.104
Noninactivating fraction %	51.9 ± 3.7	43.6 ± 4.5	0.213
*n*	9/5	15/5	–
I_Na_ parameters			
Activation V_0.5_, mV	−27.8 ± 2.2	−29.9 ± 1.2	0.363
K	3.2 ± 0.3	3.1 ± 0.2	0.806
* n*	11/6	20/7	–
ss inactivation V_0.5_, mV	−63.2 ± 3.6	−64.7 ± 1.7	0.372
K	7.8 ± 1.3	6.9 ± 0.4	0.338
*n*	5/3	16/6	–

**Figure 6:**
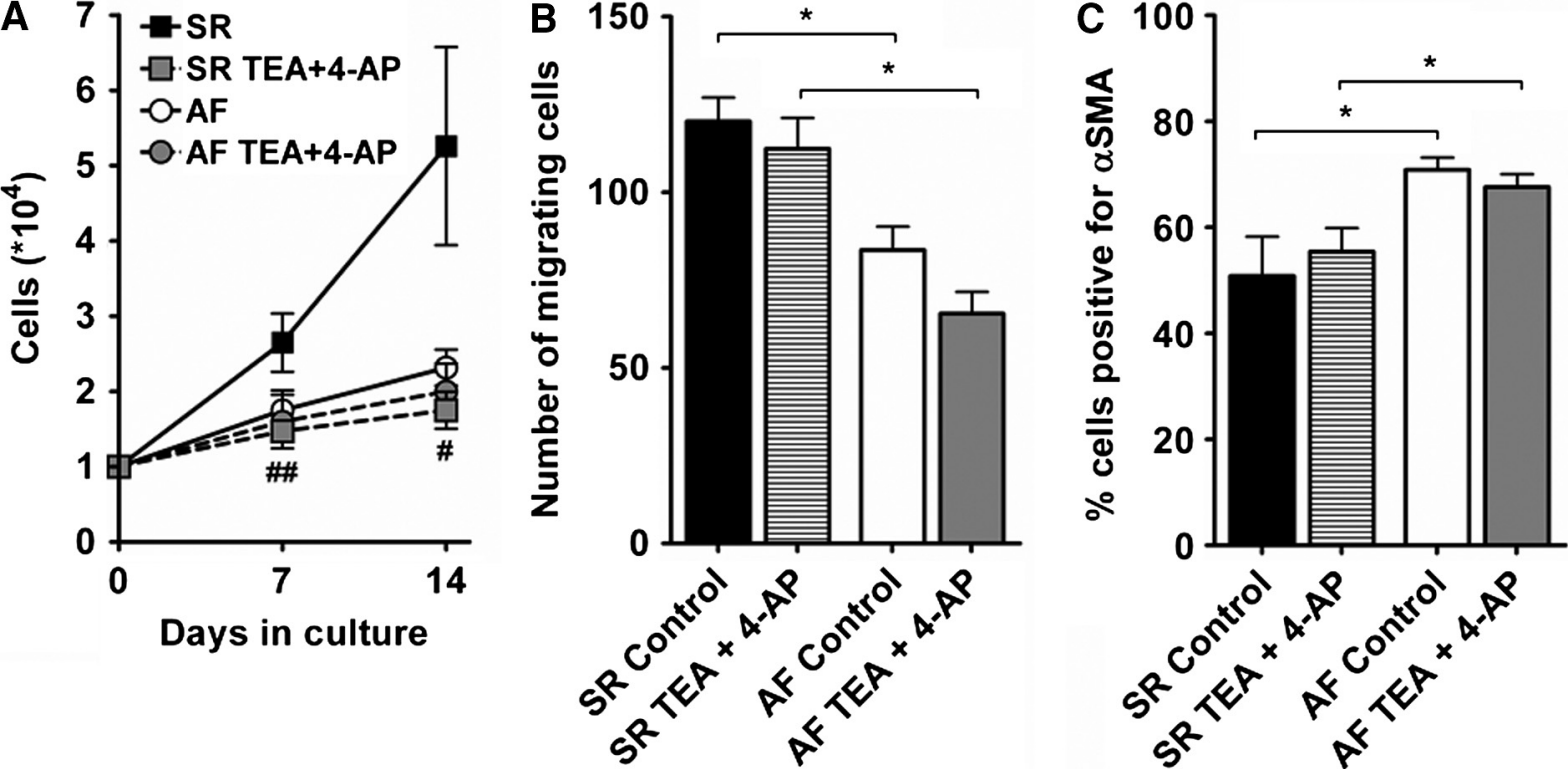
Effect of K^+^ current blockers on cellular functions of cultured fibroblasts. Fibroblasts were cultivated with normal culture medium (control) or medium supplemented with 1 mmol/L tetraethylammonium and 1 mmol/L 4‐aminopyridine (TEA + 4‐AP). Proliferation (A; SR,* n *= 10; AF,* n *= 6), migration (B; SR,* n *= 4; AF,* n *= 3), and differentiation (C; SR,* n *= 5; AF,* n *= 5) were assessed as described in Figure 2. *P < 0.05, two‐tailed unpaired Student's *t*‐test. ^#^ *P *< 0.05, ^##^ *P* < 0.01, paired Student's *t*‐test.

Voltage for half maximum activation and/or steady‐state (ss) inactivation (V_0.5_) and the respective slope factors (k) were obtained from fitting a Boltzmann function (see Material and Methods) to the data points shown in Figure 5D and Figure 6F. Data were compared using a two‐tailed unpaired Student's *t* test.

Although we found high level of transcript for KCNMA1 (Fig. 3 and Table S1), Ca^2+^‐activated potassium channels of big conductance (BKCa) did not seem to be involved, as currents were not affected by paxilline (Table 2). The nonspecific K^+^ channel blockers tetraethylammonium (TEA) and 4‐aminopyridine (4‐AP) reduced current amplitude in SR fibroblasts but did but affect currents in AF cells. This confirms the presence of voltage‐gated K^+^ (Kv) channels in SR fibroblasts and implies that those channels are absent in the AF group. These results could be explained by the significant downregulation of Kv4.1 expression and the tendency for lower transcript levels of Kv6.2 and Kv11.3 that we observed in AF fibroblasts (Fig. 3 and Table S1).

Interestingly, blocking Kv channels in culture with 1 mmol/L TEA plus 1 mmol/L 4‐AP reduced the proliferation of SR fibroblasts to the level of AF cells while it did not affect the proliferation of AF fibroblasts (Fig. 6A). The number of cells after 14 days (*10^4^) was in SR control: 5.3 ± 1.3; and in SR TEA + 4‐AP: 1.7 ± 0.2; *n* = 10; (*P* < 0.05); in AF control: 2.3 ± 0.2; and in AF TEA + 4‐AP: 2.0 ± 0.4; *n* = 6. Neither migration, nor differentiation of SR and AF fibroblasts were affected by the addition of TEA and 4‐AP in the culture medium (Fig. 6B and C).

Although the chloride channel blocker 4,4’‐diisothiocyano‐2,2’‐stilbenedisulfonic acid (DIDS) had no effect on current amplitude (Table 2) and the currents recorded were hence unlikely to be Cl^‐^ currents, we found very high levels of transcripts for various voltage sensitive Cl^−^ channels (Fig. 3 and Table S1).

### Sodium currents in cultured fibroblasts

**Figure 7:**
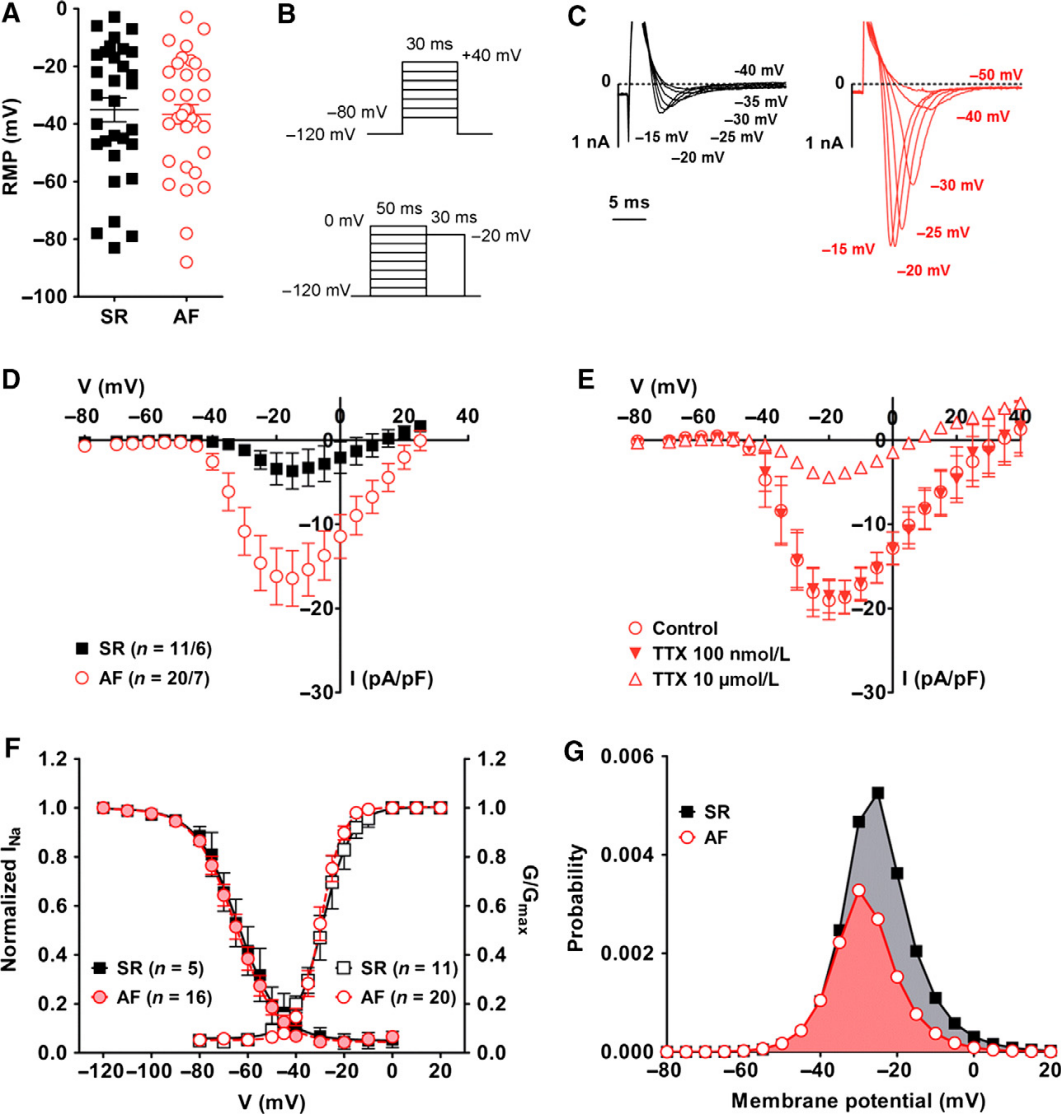
Sodium currents in cultured fibroblasts. (A) Resting membrane potential (RMP) was measured in cells from seven SR patients and six AF patients. (B) Voltage‐step protocols for current activation (top) and steady‐state inactivation (bottom). (C) Examples of currents recorded at the indicated potentials in a SR (black) and AF (red) fibroblast. (D) Current–voltage relationships. (E) Effect of 100 nmol/L and 10 *μ*mol/L tetrodotoxin (TTX) on Na^+^ currents measured in AF fibroblasts (*n *= 4/2). (F) Activation (SR: black empty squares, *n *= 11/6; AF: red empty circles, *n *= 20/7) and steady‐state inactivation (SR: black filled squares, *n *= 5/3; AF: red filled circles, *n *= 16/6) of Na^+^ currents measured in AF fibroblasts. Activation curves were calculated as normalized conductance values from the IV curves assuming a reversal potential of +78 mV. A Boltzmann function was fitted to the data points (see Material and Methods). Fitting parameters are given in Table 3. (G) Window current in SR and AF fibroblasts calculated as the product of the activation and inactivation functions (Huang et al. 2011).

SR and AF fibroblasts showed a highly variable resting membrane potential (RMP) ranging from almost 0 to −80 mV in both groups (Fig. 7A) and averaging at −35.1 ± 4.2 mV (*n* = 31/7) in SR cells, and −36.7 ± 3.4 mV (*n* = 34/6) in AF fibroblasts.

We found Na^+^ currents in fibroblasts from six of 10 SR patients and seven of nine AF patients (Fig. 7C). The presence of Na^+^ currents was not patient‐dependent since cells with and without current were measured within the same isolation. Interestingly a larger proportion of AF fibroblasts developed these currents: 57% in the AF group (28 of 49 cells) for only 32% in the SR group (13 of 41 cells). Furthermore, current amplitude was much larger in AF cells: −3.7 ± 2.1 pA/pF (*n* = 11) in SR vs. −16.4 ± 3.3 pA/pF (*n* = 20) in AF at −15 mV, *P* = 0.01, (Fig. 7D). The specific Na^+^ channel blocker tetrodotoxin (TTX) was used to confirm the nature of the currents and help identify the channel isoform involved in AF fibroblasts (Fig. 7E). A low concentration of 100 nmol/L TTX did not affect the currents, however, almost 80% of current amplitude was blocked in the presence of 10 *μ*mol/L TTX (At −15 mV: AF control, −18.7 ± 2.0 pA/pF; AF 10 *μ*mol/L TTX, −3.9 ± 0.4 pA/pF; *P* < 0.05). Similar results were obtained with TTX on SR cells (data not shown). These results strongly suggest that cardiac fibroblasts expressed the cardiac‐specific Na^+^ channel isoform Nav1.5 for which the IC_50_ of TTX is ~2 *μ*mol/L (Satin et al. 1992). On the mRNA level, the main sodium channel *α*‐subunits expressed by both SR and AF fibroblasts were Nav1.4, Nav1.5, and Nav1.7 (Fig. 3 and Table S1). The IC_50_ of TTX for Nav1.4 and Nav1.7 is in the nanomolar range (reviewed in (Catterall et al. 2005)) confirming that the currents observed were most likely conducted through the cardiac‐specific isoform Nav1.5. The main Na^+^ channel *β*‐subunits expressed was *β*_1_.

Steady‐state activation and inactivation curves are shown in Figure 7F. The parameters from curve fitting measured in AF cells were similar to those measured in SR fibroblasts (Table 3). The overlap of the activation and inactivation curves defines a voltage range (“window”) where the channels are partially activated but not fully inactivated. The probability of Na^+^ channel opening within that window was calculated as the product of the activation and inactivation functions (1/{1 + exp[(V_0.5act_‐V)/k_act_]} × 1/{1 + exp[(V−V_0.5inact_)/k_inact_]}) as described previously (Huang et al. 2011). In AF fibroblasts, we found a non‐negligible probability to have persistent opened channels between −60 mV and +20 mV with a peak value of 0.33% at −30 mV (Fig. 7G). In SR cells, peak probability reached 0.56% at −25 mV.

**Figure 8:**
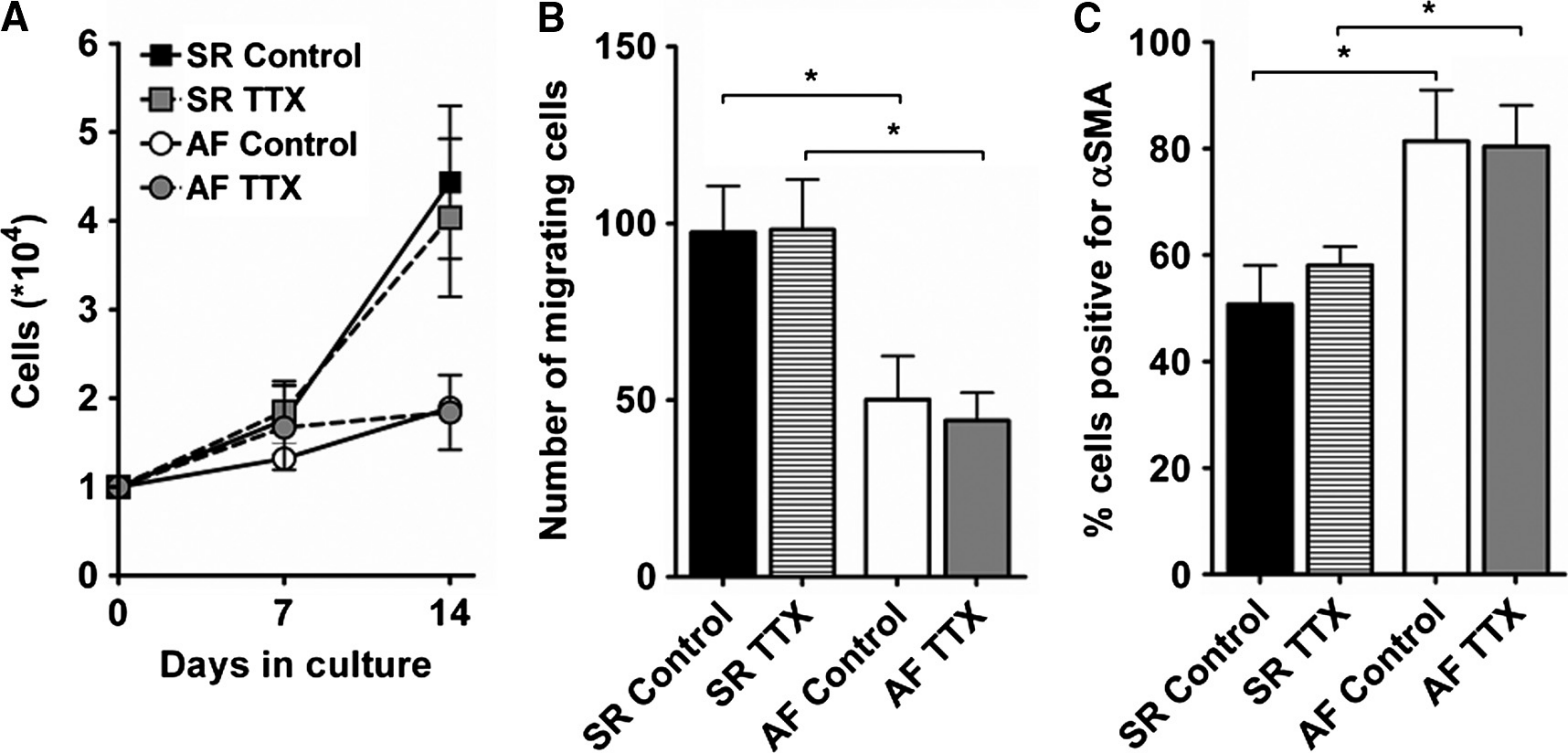
Effect of tetrodotoxin on cellular functions of cultured fibroblasts. Fibroblasts were cultivated with normal culture medium (control) or medium supplemented with 10 *μ*mol/L tetrodotoxin (TTX). Proliferation (A; SR,* n *= 7; AF,* n *= 7), migration (B; SR,* n *= 7; AF,* n *= 4) and differentiation (C; SR,* n *= 5; AF,* n *= 3) were assessed as described in Figure 2. *P < 0.05, unpaired Student's *t*‐test.

Blocking Na^+^ channels in culture with 10 *μ*mol/L TTX did not affect proliferation, migration or differentiation of SR and AF fibroblasts (Fig. 8A, B, and C).

Under the conditions used for Na^+^ and K^+^ currents measurement ([Ca^2+^]_o_^ ^= 2 mmol/L), we did not observe any inward currents with properties similar to voltage‐gated Ca^2+^ currents. Additional experiments were performed without Na^+^ in the bath solution and with 10 mmol/L Ba^2+^ as charge carrier to bypass Ca^2+^/calmodulin‐dependent inactivation of Ca^2+^ channels (Levitan 1999) We investigated the presence of functional voltage‐gated Ca^2+^ channels in 28 cells of five patients and never observed any inward currents (data not shown). It is, however, interesting to note that the expression of several calcium channel subunits, including the cardiac‐specific channel Cav1.2, was significantly upregulated in AF fibroblasts.

## Discussion

In this study, we have shown that AF fibroblasts have a reduced capacity for proliferation and migration in culture compared to SR cells. We found, however, that they differentiate more readily and develop larger Na^+^ and Kir currents than the SR group. While the involvement of Na^+^ and Kir currents in fibroblast function remains to be demonstrated, our results suggest that Kv currents regulated the proliferation of SR fibroblasts.

### Fibroblast differentiation and sodium currents

While recent studies have isolated fibroblasts by enzymatic dissociation of cardiac tissue, here we used the outgrowth technique as it enables the isolation of a larger number of cells and, in addition, is a more gentle procedure for ion channels which can be destroyed with standard enzymatic digestion (Rajamani et al. 2006). We found that significantly fewer cells migrated out of AF tissue. This could result from a reduced number of fibroblasts in the fibrillating environment; an impaired potential to migrate out of the tissue, and/or a reduced proliferative capacity once the cells are on the culture plate. Upon replating, AF fibroblasts indeed showed reduced proliferation and migration, but differentiated into myofibroblasts more readily than SR cells. A similar increase in differentiation has been previously reported for freshly isolated right atrial fibroblasts from patients with AF (Du et al. 2010). In addition, Burstein et al. showed that medium from rapidly paced cardiomyocytes considerably reduced proliferation of cultured right atrial fibroblasts and promoted their differentiation in myofibroblasts as shown by increased expression of aSMA and ECM proteins (Burstein et al. 2007). These results were corroborated by in vivo data that showed increased levels of aSMA and ECM proteins in atrial tissue of atrial‐tachypaced dogs. Taken together, these results suggest that under rapid atrial activity such as AF, cardiomyocytes produce factors that alter fibroblast function, promoting their differentiation into myofibroblasts.

An intriguing result was the development of large Na^+^ currents in more than half the population of AF fibroblasts, whereas much smaller currents were recorded in a few SR cells. TTX experiments showed that the major channel subunit responsible for these currents was the cardiac‐specific isoform Nav1.5. Activation and inactivation properties were, however, different from those reported for human ventricular and atrial myocytes as both half maximum potential for activation and inactivation were shifted toward more positive voltages (Sakakibara et al. 1993; Feng et al. 1996). This difference is most likely due to the participation of a different panel of accessory subunits. Interestingly two other groups have recently reported the expression of functional Nav1.5 channels in atrial fibroblasts (Chatelier et al. 2012; Koivumäki et al. 2014). In contrast to our study, Chatelier et al. observed Na^+^ currents in cells from SR patients after only 8 days of culture, and current density after 15 days (~13 pA/pF) was almost as big as what we observed in replated AF fibroblasts (~16 pA/pF) (Chatelier et al. 2012). Like in our experiments, the authors used right atrial appendage tissue obtained from patients undergoing cardiac bypass surgery and/or valve replacement, but a major difference, which could account for this discrepancy, was the cell isolation protocol. While we used the outgrowth technique and let fibroblasts migrate out of the explants for 3 weeks before replating; Chatelier et al. isolated fibroblasts by enzymatic digestion of the tissue. The two isolation techniques might indeed provide different populations of fibroblasts regarding their differentiation state: while the outgrowth technique selects only for fibroblasts that are able to migrate out of the tissue, and which are possibly less committed, the enzymatic digestion is likely to enable the isolation of fibroblasts at all stages of differentiation. Chatelier et al. actually correlated the presence of Na^+^ currents to the expression of aSMA and suggested that the development of such currents happens during the differentiation process. The faster appearance of Na^+^ currents in culture could therefore be explained by the presence of fibroblasts more advanced in their differentiation. This is in fact in agreement with our results that showed, in the AF group, both a larger number fibroblasts with Na^+^ currents and a larger number of cells with aSMA fibers.

The role of voltage‐gated Na^+^ channel in nonexcitable cells remains unclear. Numerous studies have demonstrated their association with cancer progression, reviewed in (Fraser et al. 2014; Patel and Brackenbury 2015). Most Na^+^ channel *α*‐subunits, including Nav1.5, were indeed shown to enhance migration and invasion of various types of cancer cells, thereby promoting metastasis. Na^+^ channels have also been linked to migration, endocytosis, and secretion of human aortic smooth muscle cells (Meguro et al. 2009), as well as angiogenesis (Andrikopoulos et al. 2011).

One of the mechanisms through which Na^+^ are likely to modulate cell function is via the activation of the Na^+^/Ca^2+^ exchanger (NCX) in reverse mode, resulting in the entry of Ca^2+^ and the subsequent activation of Ca^2+^‐dependent signaling cascades (Andrikopoulos et al. 2011). Ca^2+^ influx through NCX was indeed shown to regulate proliferation, migration, and contraction of rat ventricular fibroblasts (Raizman et al. 2007).

In this study, we did not find any connection between Na^+^ currents and proliferation, migration, or differentiation of atrial fibroblasts, and their function remains elusive. Nevertheless, a fraction of SR and AF fibroblasts had RMP below −50 mV, potentials at which Na^+^ channel are available for activation. Furthermore, at less negative potentials, Na^+^ channels are responsible for a small window current, which may be involved in other functions of fibroblasts, such as production of ECM components or secretion of cytokines. The persistent entry of Na^+^ in fibroblasts potentially coupled to cardiomyocytes might also affect cardiac electrical activity. Koivumäki et al. recently investigated this possibility using a mathematical model of fibroblast‐myocyte coupling with the membrane potential of fibroblasts set at −35 mV or −65 mV to prevent complete inactivation of Na^+^ channels (Koivumäki et al. 2014). Even at the more negative potential, introducing Na^+^ currents in the fibroblast population did not result in any significant effect on the cardiomyocyte action potential. Although further experiments are needed to confirm these observations, this work implies that if Na^+^ channels are involved in the pathophysiology of AF, it is more likely via the modulation of fibroblasts function rather than through the direct alteration of cardiomyocyte electrical activity.

### Fibroblasts proliferation and potassium currents

While we did not find any connection between Na^+^ currents and fibroblasts function, our results suggest that Kv currents are involved in the proliferation of SR fibroblasts. Such a role for Kv channels in cell cycle progression has been well documented in many other cell types, reviewed in (Blackiston et al. 2009; Urrego et al. 2014). Similar to our results with proliferation, TEA and 4‐AP blocked mitogenesis in T lymphocytes (DeCoursey et al. 1984). This differs from the more recent study by Wu et al., which showed enhanced proliferation of dog cardiac fibroblasts following incubation with TEA or 4‐AP (Wu et al. 2014).

The mechanisms through which Kv currents affect cell proliferation include modulation of RMP, which controls cell cycle progression (Blackiston et al. 2009); regulation of cell volume; and increase in driving force for Ca^2+^ which can activate downstream Ca^2+^‐dependent signaling pathways. In addition, Kv channels might act through noncanonical mechanisms involving direct interactions with members of signaling cascades (Hegle et al. 2006).

Although Kv current density was similar in SR and AF fibroblasts, TEA and 4‐AP reduced K^+^ currents in SR cells only. This suggests a modulation of Kv channels expression and/or regulation in AF fibroblasts, which might be the cause for the altered physiological functions we observed in AF cells, especially the reduced proliferation. Our gene expression data identified three candidates, Kv4.1, Kv6.2, and Kv11.3, with reduced expression in AF fibroblasts. Further experiments are needed to investigate the importance of these specific channel subunits in the regulation of fibroblasts proliferation.

Through their modulation of RMP, Kir channels have long been linked to physiological functions of cardiac fibroblasts, including proliferation and contractility (Chilton et al. 2005). A recent study by Qi et al. showed that freshly isolated left atrial fibroblasts from a dog model of CHF display enhanced proliferation and differentiation, larger Ba^2+^‐sensitive Kir currents, and a more negative RMP than control cells (Qi et al. 2015). They identified KCNJ2 as the likely candidate for these changes and demonstrated that KCNJ2 expression modulates RMP and cell cycle progression. In this study, significantly larger inward rectifier currents were observed in cultured AF fibroblasts compared to cultured SR cells. While current density was similar to that observed in dog atrial fibroblasts (Qi et al. 2015), currents were not sensitive to Ba^2+^ suggesting that different channels are involved. Kir channels insensitive to Ba^2+^ include the K_ATP_ subunits Kir6.1 and Kir6.2, as well as Kir3.3 and Kir7.1 which are involved in setting the resting membrane potential of various cell types other than cardiac cells (Kubo et al. 2005). Interestingly, Kir6.1 activation was shown to be associated with increased proliferation of murine ventricular fibroblasts and reduced secretion of interleukin 6 (Benamer et al. 2009). While we detected moderate levels of transcripts for Kir3.3, Kir6.1, and Kir6.2, the expression level for Kir7.1 was very low. Further experiments with other specific inhibitors, such as the K_ATP_ channel blocker glibenclamide, are needed to identify the currents observed in SR and AF fibroblasts.

The importance of fibroblast Kir currents on cardiac electrical activity has been highlighted in a recent study (Aguilar et al. 2014). Using a mathematical model of cardiomyocyte‐fibroblast coupling, Aguilar et al. indeed showed that upregulation of Kir currents in fibroblasts has profibrillatory consequences due mainly to shortening of action potential duration.

Another group of channels which might have been responsible for the ion currents observed in fresh and replated fibroblasts is the TRP channel family. These nonselective cation channels have recently aroused much interest in the context of fibrogenesis. Four members, TRPC3, TRPC6, TRPM7, and TRPV4 were shown to be involved in (TGFB1‐induced) activation of fibroblasts, most likely through calcium influx and the subsequent activation of calcium‐dependent downstream signaling pathways (Du et al. 2010; Davis et al. 2012; Harada et al. 2012; Adapala et al. 2013). In addition, TRPC3 and TRPM7 were upregulated in AF fibroblasts, and might thus contribute to the pathophysiology of the disease (Du et al. 2010; Harada et al. 2012). In our study, however, the expression of TRPC3 and TRPM7 was similar between SR and AF samples, a discrepancy that might result from differences in culture conditions (see below).

Other ion channels that have been linked to proliferation and differentiation of cardiac fibroblasts include BKCa and the H^+^/Cl^‐^ transporter CLCN3 which were shown to regulate cell cycle progression in human ventricular fibroblasts (He et al. 2011). It was also recently suggested that the calcium activated Cl^‐^ channel ANO1 contributes to angiotensin II‐induced activation of fibroblasts (El Chemaly et al. 2014). While we did not find any functional evidence for the presence of such currents in our fibroblasts, we found high levels of transcripts for the corresponding channel subunits, ANO1 being significantly upregulated in AF cells.

### Differences between fresh and cultured fibroblasts

Culture time strongly influences fibroblasts properties. Using a dog model of CHF, Dawson et al. compared fresh and cultured atrial fibroblasts (Dawson et al. 2012). They reported that while they observed significant differences between fresh control and diseased cells regarding expression of ECM proteins and amplitude of K^+^ currents, these differences disappeared after only 48 h of culture. In this study, we compared ion currents in fresh and replated fibroblasts using a voltage‐ramp from −120 to 40 mV. We found that ion currents in fresh cells were weak rectifiers, with a reversal potential at 0 mV, possibly conducted through leak or TRP channels. In replated fibroblasts, current amplitude was largely diminished but half of the cells showed outward rectifier currents that activated at −20 mV. Our results therefore confirm that ion currents are extensively remodeled when fibroblasts are kept in culture and further highlight the caution needed when extrapolating data obtained from cells kept in such conditions. For instance, we and others have observed the development of voltage‐gated Na^+^ currents together with differentiation in myofibroblasts in vitro, but whether this phenomenon also occurs in vivo and is not only an artifact of culture conditions is a key point that needs to be addressed.

Nevertheless, we observed differences between cultured SR and AF fibroblasts up to 5 weeks after isolation, implying that their phenotype had been affected not only by culture time but also by disease conditions. The fibrillating environment induced changes in atrial fibroblasts, the consequences of which were revealed in our artificial system. As mentioned above, these changes might include reduced proliferative capacity and/or increased differentiation into myofibroblasts, which could exhaust the pool of fibroblasts available in the heart. Further experiments are needed to investigate these points, for instance characterizing fibroblast/myofibroblast populations in SR and AF tissue sections would provide an insight into the impact of atrial fibrillation on atrial fibroblasts.

### Limitations

The use of pharmacological agents in culture medium to investigate the role of ion currents in major cell functions is a limitation in this study. We cannot exclude that the effects observed were due to off‐target actions of the drugs. In addition, ion channels might act through noncanonical mechanisms involving direct interactions with downstream signaling partners. Downregulating specifically the expression of ion channels, using siRNA for instance, would therefore provide valuable information on their involvement in fibroblasts function as well as their mechanism of action.

Microarrays are a great tool to assess the expression of a substantial number of genes but measurement of mRNA levels is less accurate than with quantitative PCR which is a more sensitive technique. The main purpose of our gene expression profiling is to give directions for further investigations and any difference, or suspicion of difference, in mRNA levels between SR and AF should therefore be confirmed by quantitative PCR.

## Conclusions

Cardiac fibroblasts are a primordial component of the heart as they support cardiac function by ensuring the integrity of the tissue. But excessive fibroblast activity under pathological conditions can lead to detrimental fibrosis and be involved in cardiac dysfunction. This study shows that in the context of chronic atrial fibrillation, fibroblasts undergo phenotypic changes that are revealed in culture. While their capacity for proliferation and migration is impaired, their differentiation in myofibroblasts is increased. This is accompanied by changes in electrophysiological properties, which might modulate physiological functions and alter cardiac electrical activity; and thereby contribute to the pathophysiology of the disease.

## Conflict of interest

None declared.

## Supporting information




**Table S1.** Gene expression of major ECM components, growth factors, membrane proteins and ion channels in cultured fibroblasts.
