# Supplementary material for: Altered physiological functions and ion currents in atrial fibroblasts from patients with chronic atrial fibrillation
Source: Physiol Rep. 2016 Jan 26;4(2):e12681. doi: 10.14814/phy2.12681 (PMC4760386; doi:10.14814/phy2.12681)
Supplement: Supplementary file 1 — Table S1. Gene expression of major ECM components, growth factors, membrane proteins and ion channels in cultured fibroblasts. [file PHY2-4-e12681-s001.docx]

**Supplementary Table 1. Gene expression of major ECM components, growth factors, membrane proteins and ion channels in cultured fibroblasts.** Boxes denote significant (double line, p < 0.05) and non-significant (dotted line, 0.05 < p < 0.1) differences between SR and AF.

| **Gene** | **Expression levels 2^[mean]^** | | **Fold-change AF vs. SR** | ***p values*** |
| --- | --- | --- | --- | --- |
|  | **SR** | **AF** |  |  |
| **ECM Proteins** |  |  |  |  |
| **ELN** | 470 | 470 | 1.00 | *0.890* |
| **FN1** | 16973 | 16891 | 1.00 | *0.613* |
| **COL1A1** | 10599 | 10128 | -1.05 | *0.342* |
| **COL1A2** | 10670 | 10495 | -1.03 | *0.679* |
| **COL2A1** | 317 | 299 | -1.07 | *0.257* |
| **COL3A1** | 3598 | 3356 | -1.08 | *0.417* |
| **COL4A1** | 3587 | 3772 | 1.05 | *0.632* |
| **COL4A2** | 3725 | 3749 | 1.01 | *0.924* |
| **COL4A3** | 184 | 175 | -1.05 | *0.450* |
| **COL4A4** | 194 | 194 | -1.00 | *0.990* |
| **COL4A5** | 145 | 142 | -1.02 | *0.777* |
| **COL4A6** | 82 | 74 | -1.12 | *0.025* |
| **COL5A1** | 983 | 1058 | 1.08 | *0.240* |
| **COL5A2** | 1153 | 1226 | 1.06 | *0.407* |
| **COL5A3** | 249 | 254 | 1.03 | *0.520* |
| **COL6A1** | 4365 | 4518 | 1.03 | *0.550* |
| **COL6A2** | 2703 | 2885 | 1.07 | *0.360* |
| **COL6A3** | 3687 | 3570 | -1.03 | *0.848* |
| **COL6A6** | 178 | 162 | -1.10 | *0.665* |
| **COL7A1** | 216 | 213 | -1.01 | *0.908* |
| **COL8A1** | 293 | 299 | -1.00 | *0.994* |
| **COL8A2** | 265 | 264 | 1.01 | *0.895* |
| **COL9A1** | 112 | 105 | -1.08 | *0.226* |
| **COL9A2** | 203 | 205 | 1.00 | *0.960* |
| **COL9A3** | 398 | 362 | -1.09 | *0.137* |
| **COL10A1** | 83 | 73 | -1.13 | *0.013* |
| **COL11A1** | 118 | 108 | -1.08 | *0.455* |
| **COL11A2** | 199 | 192 | -1.00 | *0.959* |
| **COL12A1** | 1541 | 1771 | 1.16 | *0.370* |
| **COL13A1** | 199 | 209 | 1.03 | *0.634* |
| **COL14A1** | 135 | 150 | 1.10 | *0.551* |
| **COL15A1** | 476 | 724 | 1.52 | *0.089* |
| **COL16A1** | 326 | 321 | -1.04 | *0.700* |
| **COL17A1** | 129 | 132 | 1.03 | *0.608* |
| **COL18A1** | 387 | 388 | 1.01 | *0.808* |
| **COL19A1** | 71 | 70 | -1.03 | *0.387* |
| **COL20A1** | 188 | 187 | -1.00 | *0.994* |
| **COL21A1** | 92 | 89 | -1.01 | *0.777* |
| **COL22A1** | 172 | 167 | -1.02 | *0.660* |
| **COL23A1** | 322 | 305 | -1.05 | *0.243* |
| **COL24A1** | 62 | 58 | -1.02 | *0.638* |
| **COL25A1** | 128 | 122 | -1.05 | *0.408* |
| **COL27A1** | 254 | 249 | -1.03 | *0.537* |
| **COL28A1** | 61 | 58 | -1.06 | *0.265* |
| **COL29A1** | 20 | 21 | 1.04 | *0.352* |
| **LAMA1** | 571 | 579 | 1.02 | *0.908* |
| **LAMA2** | 1078 | 1296 | 1.21 | *0.166* |
| **LAMA3** | 141 | 167 | 1.18 | *0.173* |
| **LAMA4** | 426 | 529 | 1.27 | *0.346* |
| **LAMA5** | 375 | 375 | 1.01 | *0.887* |
| **LAMB1** | 3819 | 3681 | -1.03 | *0.680* |
| **LAMB2** | 1721 | 1750 | 1.02 | *0.795* |
| **LAMB3** | 115 | 120 | 1.05 | *0.406* |
| **LAMB4** | 51 | 48 | -1.07 | *0.105* |
| **LAMC1** | 4234 | 4392 | 1.03 | *0.626* |
| **LAMC2** | 102 | 140 | 1.34 | *0.352* |
| **LAMC3** | 227 | 214 | -1.07 | *0.137* |
| **MMP1** | 899 | 326 | -2.81 | *0.085* |
| **MMP2** | 10033 | 9878 | -1.01 | *0.795* |
| **MMP3** | 47 | 48 | 1.03 | *0.459* |
| **MMP7** | 20 | 22 | 1.06 | *0.148* |
| **MMP8** | 26 | 21 | -1.22 | *0.290* |
| **MMP9** | 103 | 113 | 1.08 | *0.110* |
| **MMP10** | 39 | 39 | 1.01 | *0.806* |
| **MMP11** | 385 | 401 | 1.04 | *0.798* |
| **MMP12** | 27 | 29 | 1.06 | *0.451* |
| **MMP13** | 37 | 36 | -1.04 | *0.380* |
| **MMP14** | 3469 | 3433 | -1.02 | *0.845* |
| **MMP15** | 250 | 244 | -1.06 | *0.230* |
| **MMP16** | 137 | 147 | 1.08 | *0.752* |
| **MMP17** | 239 | 237 | -1.02 | *0.724* |
| **MMP19** | 190 | 174 | -1.07 | *0.208* |
| **MMP20** | 58 | 58 | -1.01 | *0.875* |
| **MMP21** | 94 | 86 | -1.08 | *0.188* |
| **MMP23B** | 511 | 476 | -1.08 | *0.141* |
| **MMP24** | 250 | 266 | 1.05 | *0.370* |
| **MMP25** | 120 | 111 | -1.06 | *0.248* |
| **MMP26** | 25 | 24 | -1.02 | *0.769* |
| **MMP27** | 39 | 38 | -1.04 | *0.418* |
| **MMP28** | 187 | 189 | 1.01 | *0.810* |
| **TIMP1** | 13237 | 13820 | 1.05 | *0.578* |
| **TIMP2** | 6173 | 6205 | 1.01 | *0.835* |
| **TIMP3** | 6288 | 6169 | -1.01 | *0.927* |
| **TIMP4** | 130 | 126 | -1.04 | *0.409* |
|  |  |  |  |  |
| **Growth Factors** |  |  |  |  |
| **CTGF** | 10204 | 10508 | 1.03 | *0.589* |
| **EGF** | 56 | 55 | -1.04 | *0.452* |
| **EGFR** | 1024 | 1029 | 1.02 | *0.848* |
| **FGF1** | 373 | 456 | 1.21 | *0.056* |
| **FGF2** | 1776 | 2105 | 1.17 | *0.163* |
| **FGF3** | 157 | 155 | -1.01 | *0.925* |
| **FGF4** | 291 | 278 | -1.04 | *0.423* |
| **FGF5** | 464 | 569 | 1.20 | *0.480* |
| **FGF6** | 81 | 78 | -1.04 | *0.499* |
| **FGF7** | 553 | 969 | 1.74 | *0.025* |
| **FGF8** | 192 | 173 | -1.09 | *0.307* |
| **FGF9** | 68 | 102 | 1.50 | *0.166* |
| **FGF10** | 48 | 46 | -1.06 | *0.123* |
| **FGF11** | 251 | 259 | 1.02 | *0.869* |
| **FGF12** | 69 | 66 | -1.03 | *0.596* |
| **FGF13** | 97 | 97 | 1.07 | *0.280* |
| **FGF14** | 42 | 40 | -1.02 | *0.767* |
| **FGF16** | 44 | 43 | -1.05 | *0.634* |
| **FGF17** | 170 | 165 | -1.02 | *0.731* |
| **FGF18** | 199 | 205 | 1.00 | *0.963* |
| **FGF19** | 117 | 116 | -1.01 | *0.906* |
| **FGF20** | 113 | 114 | 1.01 | *0.871* |
| **FGF21** | 78 | 74 | -1.05 | *0.296* |
| **FGF22** | 281 | 264 | -1.06 | *0.172* |
| **FGF23** | 102 | 97 | -1.06 | *0.403* |
| **FGFR1** | 1229 | 1330 | 1.07 | *0.392* |
| **FGFR2** | 127 | 129 | 1.00 | *0.991* |
| **FGFR3** | 150 | 151 | -1.03 | *0.562* |
| **FGFR4** | 119 | 110 | -1.11 | *0.122* |
| **IGF1** | 72 | 94 | 1.26 | *0.157* |
| **IGF1R** | 1317 | 1368 | 1.03 | *0.591* |
| **IGF2** | 224 | 151 | -1.50 | *0.277* |
| **IGF2R** | 2831 | 2998 | 1.06 | *0.292* |
| **PDGFA** | 605 | 915 | 1.52 | *0.005* |
| **PDGFB** | 195 | 198 | 1.03 | *0.777* |
| **PDGFC** | 414 | 452 | 1.08 | *0.556* |
| **PDGFD** | 465 | 571 | 1.22 | *0.334* |
| **PDGFRA** | 1953 | 2104 | 1.07 | *0.401* |
| **PDGFRB** | 1053 | 1130 | 1.09 | *0.681* |
| **TGFA** | 28 | 29 | 1.02 | *0.688* |
| **TGFB1** | 1230 | 1396 | 1.13 | *0.059* |
| **TGFB1I1** | 1784 | 1750 | -1.02 | *0.863* |
| **TGFB2** | 4929 | 4593 | -1.08 | *0.431* |
| **TGFB3** | 276 | 312 | 1.12 | *0.522* |
| **TGFBI** | 4430 | 4907 | 1.11 | *0.245* |
| **TGFBR1** | 3232 | 3486 | 1.08 | *0.519* |
| **TGFBR2** | 1385 | 1392 | 1.01 | *0.951* |
| **TGFBR3** | 197 | 206 | 1.06 | *0.694* |
| **VEGFA** | 2618 | 3025 | 1.15 | *0.186* |
| **VEGFB** | 909 | 964 | 1.07 | *0.652* |
| **VEGFC** | 1090 | 1355 | 1.23 | *0.079* |
|  |  |  |  |  |
| **Membrane Proteins** | |  |  |  |
| **GJA1 (Cx43)** | 6041 | 5416 | -1.11 | 0.159 |
| **GJA3 (Cx46)** | 179 | 161 | -1.10 | 0.141 |
| **GJA4 (Cx37)** | 203 | 197 | -1.03 | 0.624 |
| **GJA5 (Cx40)** | 45 | 49 | 1.11 | 0.658 |
| **GJA8 (Cx50)** | 79 | 79 | -1.03 | 0.537 |
| **GJA10 (Cx58)** | 50 | 53 | 1.07 | 0.197 |
| **GJB1 (Cx32)** | 50 | 51 | 1.02 | 0.602 |
| **GJB2 (Cx26)** | 51 | 47 | -1.08 | 0.032 |
| **GJB3 (Cx31)** | 74 | 72 | 1.02 | 0.684 |
| **GJB4 (Cx30.3)** | 95 | 97 | 1.04 | 0.507 |
| **GJB5 (Cx31.1)** | 104 | 96 | -1.09 | 0.146 |
| **GJB6 (Cx30)** | 60 | 56 | -1.09 | 0.208 |
| **GJB7 (Cx25)** | 23 | 22 | 1.02 | 0.738 |
| **GJC1 (Cx45)** | 298 | 321 | 1.11 | 0.404 |
| **GJC2 (Cx47)** | 583 | 572 | -1.02 | 0.718 |
| **GJC3 (Cx30.2)** | 69 | 73 | 1.03 | 0.548 |
| **GJD2 (Cx36)** | 257 | 263 | 1.03 | 0.503 |
| **GJD3 (Cx31.9)** | 611 | 563 | -1.08 | 0.150 |
| **GJD4 (Cx39)** | 112 | 107 | -1.04 | 0.425 |
| **ITGA2** | 2594 | 2812 | 1.08 | 0.614 |
| **ITGA2B** | 95 | 88 | -1.05 | 0.452 |
| **ITGA3** | 2600 | 2623 | 1.01 | 0.831 |
| **ITGA4** | 682 | 798 | 1.17 | 0.472 |
| **ITGA5** | 5653 | 5468 | -1.03 | 0.576 |
| **ITGA6** | 252 | 223 | -1.13 | 0.494 |
| **ITGA7** | 549 | 795 | 1.45 | 0.055 |
| **ITGA8** | 1338 | 1851 | 1.39 | 0.125 |
| **ITGA9** | 127 | 124 | -1.04 | 0.428 |
| **ITGA10** | 91 | 82 | -1.11 | 0.066 |
| **ITGA11** | 5418 | 5783 | 1.08 | 0.474 |
| **ITGAD** | 146 | 141 | -1.06 | 0.104 |
| **ITGAE** | 125 | 119 | -1.06 | 0.173 |
| **ITGAL** | 61 | 63 | -1.01 | 0.917 |
| **ITGAM** | 101 | 107 | 1.04 | 0.392 |
| **ITGAV** | 5525 | 5647 | 1.01 | 0.917 |
| **ITGAX** | 102 | 103 | 1.02 | 0.722 |
| **ITGB1** | 7284 | 7272 | -1.00 | 0.939 |
| **ITGB2** | 150 | 152 | 1.02 | 0.827 |
| **ITGB3** | 993 | 1087 | 1.08 | 0.727 |
| **ITGB4** | 108 | 106 | -1.01 | 0.814 |
| **ITGB5** | 4713 | 5076 | 1.09 | 0.235 |
| **ITGB6** | 51 | 52 | -1.00 | 0.944 |
| **ITGB7** | 112 | 112 | 1.01 | 0.867 |
| **ITGB8** | 2214 | 2093 | -1.07 | 0.520 |
| **CDH1** | 111 | 123 | 1.12 | 0.062 |
| **CDH2** | 5866 | 5879 | 1.00 | 0.980 |
| **CDH3** | 89 | 93 | 1.02 | 0.685 |
| **CDH4** | 231 | 219 | -1.05 | 0.542 |
| **CDH5** | 270 | 243 | -1.12 | 0.718 |
| **CDH6** | 2794 | 3078 | 1.11 | 0.637 |
| **CDH7** | 34 | 33 | -1.04 | 0.366 |
| **CDH8** | 486 | 264 | -1.86 | 0.057 |
| **CDH9** | 26 | 26 | 1.05 | 0.106 |
| **CDH10** | 297 | 200 | -1.47 | 0.091 |
| **CDH11** | 7882 | 7835 | -1.00 | 0.969 |
| **CDH12** | 29 | 28 | -1.04 | 0.374 |
| **CDH13** | 4399 | 5298 | 1.20 | 0.040 |
| **CDH15** | 145 | 134 | -1.07 | 0.186 |
| **CDH16** | 93 | 89 | -1.02 | 0.668 |
| **CDH17** | 32 | 30 | -1.08 | 0.084 |
| **CDH18** | 33 | 32 | -1.01 | 0.806 |
| **CDH19** | 50 | 74 | 1.50 | 0.004 |
| **CDH20** | 83 | 84 | -1.01 | 0.874 |
| **CDH29** | 108 | 103 | -1.04 | 0.317 |
|  |  |  |  |  |
| **Ion Channels** |  |  |  |  |
| **KCNJ1 (Kir1.1)** | 18 | 19 | 1.02 | *0.825* |
| **KCNJ2 (Kir2.1)** | 93 | 89 | -1.04 | *0.475* |
| **KCNJ12 (Kir2.2)** | 240 | 235 | -1.01 | *0.806* |
| **KCNJ4 (Kir2.3)** | 200 | 198 | -1.01 | *0.842* |
| **KCNJ14 (Kir2.4)** | 96 | 93 | -1.03 | *0.655* |
| **KCNJ3 (Kir3.1)** | 80 | 78 | -1.03 | *0.595* |
| **KCNJ6 (Kir3.2)** | 42 | 45 | 1.04 | *0.406* |
| **KCNJ9 (Kir3.3)** | 132 | 124 | -1.06 | *0.468* |
| **KCNJ5 (Kir3.4)** | 114 | 114 | 1.01 | *0.939* |
| **KCNJ10 (Kir4.1)** | 55 | 54 | -1.05 | *0.356* |
| **KCNJ15 (Kir4.2)** | 67 | 69 | 1.02 | *0.633* |
| **KCNJ16 (Kir5.1)** | 32 | 32 | 1.03 | *0.634* |
| **KCNJ8 (Kir6.1)** | 126 | 119 | -1.07 | *0.544* |
| **KCNJ11 (Kir6.2)** | 99 | 99 | 1.01 | *0.924* |
| **KCNJ13 (Kir7.1)** | 19 | 19 | -1.00 | *0.945* |
| **KCNMA1 (BK)** | 482 | 573 | 1.19 | *0.224* |
| **KCNN1 (SK1)** | 86 | 86 | 1.00 | *0.968* |
| **KCNN2 (SK2)** | 100 | 101 | 1.03 | *0.305* |
| **KCNN3 (SK3)** | 97 | 94 | -1.02 | *0.708* |
| **KCNN4 (SK4)** | 154 | 167 | 1.09 | *0.202* |
| **KCNA1 (Kv1.1)** | 117 | 120 | 1.02 | *0.679* |
| **KCNA2 (Kv1.2)** | 73 | 73 | -1.01 | *0.799* |
| **KCNA3 (Kv1.3)** | 68 | 81 | 1.17 | *0.156* |
| **KCNA4 (Kv1.4)** | 91 | 93 | 1.05 | *0.194* |
| **KCNA5 (Kv1.5)** | 90 | 100 | 1.10 | *0.120* |
| **KCNA6 (Kv1.6)** | 69 | 68 | 1.00 | *0.977* |
| **KCNA7 (Kv1.7)** | 74 | 68 | -1.07 | *0.133* |
| **KCNA10 (Kv1.8)** | 137 | 134 | -1.04 | *0.476* |
| **KCNB1 (Kv2.1)** | 122 | 120 | 1.04 | *0.553* |
| **KCNB2 (Kv2.2)** | 67 | 63 | -1.07 | *0.191* |
| **KCNC1 (Kv3.1)** | 221 | 215 | -1.03 | *0.651* |
| **KCNC2 (Kv3.2)** | 95 | 93 | -1.03 | *0.506* |
| **KCNC3 (Kv3.3)** | 242 | 236 | -1.01 | *0.741* |
| **KCNC4 (Kv3.4)** | 310 | 335 | 1.05 | *0.420* |
| **KCND1 (Kv4.1)** | 103 | 90 | -1.18 | *0.031* |
| **KCND2 (Kv4.2)** | 87 | 82 | -1.06 | *0.725* |
| **KCND3 (Kv4.3)** | 239 | 309 | 1.31 | *0.163* |
| **KCNF1 (Kv5.1)** | 242 | 233 | -1.05 | *0.475* |
| **KCNG1 (Kv6.1)** | 283 | 298 | 1.04 | *0.733* |
| **KCNG2 (Kv6.2)** | 367 | 338 | -1.10 | *0.091* |
| **KCNG3 (Kv6.3)** | 70 | 68 | -1.05 | *0.483* |
| **KCNG4 (Kv6.4)** | 135 | 128 | -1.04 | *0.619* |
| **KCNQ1 (Kv7.1)** | 120 | 111 | -1.05 | *0.272* |
| **KCNQ2 (Kv7.2)** | 99 | 97 | -1.00 | *0.986* |
| **KCNQ3 (Kv7.3)** | 130 | 122 | -1.09 | *0.221* |
| **KCNQ4 (Kv7.4)** | 106 | 104 | -1.01 | *0.934* |
| **KCNQ5 (Kv7.5)** | 438 | 483 | 1.11 | *0.404* |
| **KCNS1 (Kv9.1)** | 62 | 66 | 1.07 | *0.362* |
| **KCNS2 (Kv9.2)** | 131 | 136 | 1.04 | *0.367* |
| **KCNS3 (Kv9.3)** | 100 | 104 | 1.06 | *0.175* |
| **KCNH1 (Kv10.1)** | 52 | 50 | -1.01 | *0.688* |
| **KCNH5 (Kv10.2)** | 34 | 36 | 1.03 | *0.371* |
| **KCNH2 (Kv11.1)** | 174 | 166 | -1.05 | *0.334* |
| **KCNH6 (Kv11.2)** | 112 | 113 | 1.00 | *0.990* |
| **KCNH7 (Kv11.3)** | 42 | 39 | -1.08 | *0.058* |
| **KCNH8 (Kv12.1)** | 36 | 36 | -1.02 | *0.156* |
| **KCNH3 (Kv12.2)** | 176 | 166 | -1.05 | *0.314* |
| **KCNH4 (Kv12.3)** | 200 | 196 | -1.03 | *0.563* |
| **SCN1A (Nav1.1)** | 25 | 24 | -1.07 | *0.157* |
| **SCN2A (Nav1.2)** | 46 | 31 | -1.42 | *0.082* |
| **SCN3A (Nav1.3)** | 54 | 43 | -1.25 | *0.153* |
| **SCN4A (Nav1.4)** | 180 | 173 | -1.02 | *0.707* |
| **SCN5A (Nav1.5)** | 183 | 213 | 1.14 | *0.450* |
| **SCN7A (Nav2.1)** | 21 | 20 | -1.04 | *0.609* |
| **SCN8A (Nav1.6)** | 122 | 113 | -1.06 | *0.565* |
| **SCN9A (Nav1.7)** | 205 | 187 | -1.13 | *0.603* |
| **SCN10A (Nav1.8)** | 52 | 53 | -1.00 | *0.918* |
| **SCN11A (Nav1.9)** | 52 | 50 | -1.02 | *0.641* |
| **SCN1B (NavB1)** | 220 | 228 | 1.02 | *0.626* |
| **SCN2B (NavB2)** | 92 | 90 | -1.02 | *0.808* |
| **SCN3B (NavB3)** | 102 | 102 | -1.02 | *0.653* |
| **SCN4B (NavB4)** | 147 | 145 | -1.01 | *0.756* |
| **CACNA1S (Cav1.1)** | 101 | 103 | 1.02 | *0.677* |
| **CACNA1C (Cav1.2)** | 133 | 168 | 1.26 | *0.015* |
| **CACNA1D (Cav1.3)** | 64 | 60 | -1.08 | *0.059* |
| **CACNA1F (Cav1.4)** | 89 | 83 | -1.06 | *0.195* |
| **CACNA1A (Cav2.1)** | 107 | 100 | -1.08 | *0.116* |
| **CACNA1B (Cav2.2)** | 128 | 131 | 1.02 | *0.583* |
| **CACNA1E (Cav2.3)** | 81 | 81 | 1.01 | *0.868* |
| **CACNA1G (Cav3.1)** | 125 | 124 | -1.03 | *0.401* |
| **CACNA1H (Cav3.2)** | 194 | 211 | 1.09 | *0.140* |
| **CACNA1I (Cav3.3)** | 133 | 138 | 1.02 | *0.487* |
| **CACNB1 (CavB1)** | 418 | 418 | 1.02 | *0.700* |
| **CACNB2 (CavB2)** | 97 | 84 | -1.17 | *0.010* |
| **CACNB3 (CavB3)** | 601 | 576 | -1.05 | *0.543* |
| **CACNB4 (CavB4)** | 419 | 603 | 1.46 | *0.035* |
| **HCN1** | 60 | 50 | -1.19 | *0.176* |
| **HCN2** | 222 | 227 | 1.03 | *0.531* |
| **HCN3** | 195 | 183 | -1.03 | *0.475* |
| **HCN4** | 243 | 238 | -1.04 | *0.508* |
| **ANO1** | 148 | 235 | 1.57 | *0.009* |
| **CLCN1** | 78 | 83 | 1.09 | *0.148* |
| **CLCN2** | 184 | 180 | -1.04 | *0.346* |
| **CLCN3** | 802 | 803 | 1.01 | *0.890* |
| **CLCN4** | 145 | 161 | 1.12 | *0.432* |
| **CLCN5** | 628 | 661 | 1.05 | *0.494* |
| **CLCN6** | 664 | 676 | 1.02 | *0.682* |
| **CLCN7** | 1532 | 1582 | 1.03 | *0.533* |
| **CLCNKA** | 115 | 107 | -1.05 | *0.561* |
| **CLCNKB** | 100 | 110 | 1.09 | *0.105* |
| **TRPA1** | 79 | 79 | -1.01 | *0.947* |
| **TRPC1** | 348 | 362 | 1.05 | *0.560* |
| **TRPC2** | 85 | 88 | 1.04 | *0.405* |
| **TRPC3** | 42 | 41 | -1.01 | *0.760* |
| **TRPC4** | 114 | 118 | 1.04 | *0.857* |
| **TRPC5** | 55 | 52 | -1.05 | *0.319* |
| **TRPC6** | 31 | 31 | 1.02 | *0.733* |
| **TRPC7** | 31 | 31 | 1.03 | *0.606* |
| **TRPM1** | 74 | 69 | -1.08 | *0.050* |
| **TRPM2** | 148 | 143 | -1.06 | *0.393* |
| **TRPM3** | 108 | 99 | -1.12 | *0.362* |
| **TRPM4** | 436 | 434 | -1.00 | *0.961* |
| **TRPM5** | 158 | 150 | -1.03 | *0.707* |
| **TRPM6** | 43 | 44 | 1.01 | *0.707* |
| **TRPM7** | 961 | 974 | -1.00 | *0.990* |
| **TRPM8** | 41 | 39 | -1.07 | *0.310* |
| **TRPV1** | 216 | 206 | -1.06 | *0.169* |
| **TRPV2** | 720 | 750 | 1.04 | *0.421* |
| **TRPV3** | 91 | 96 | 1.06 | *0.290* |
| **TRPV4** | 146 | 150 | 1.03 | *0.631* |
| **TRPV5** | 74 | 71 | -1.06 | *0.248* |
| **TRPV6** | 43 | 45 | 1.02 | *0.635* |
